# Introducing biosimilar competition for cell and gene therapy products

**DOI:** 10.1093/jlb/lsae015

**Published:** 2024-07-15

**Authors:** Brian Canter, Sabine Sussman, Stephen Colvill, Nitzan Arad, Elizabeth Staton, Arti Rai

**Affiliations:** Duke University Margolis Institute for Health Policy, Durham, NC 27708, USA; Duke University Margolis Institute for Health Policy, Durham, NC 27708, USA; Duke University Margolis Institute for Health Policy, Durham, NC 27708, USA; Duke University Margolis Institute for Health Policy, Durham, NC 27708, USA; Duke University Margolis Institute for Health Policy, Durham, NC 27708, USA; Duke University Margolis Institute for Health Policy, Durham, NC 27708, USA; Center for Innovation Policy, Duke University School of Law, Durham, NC 27708, USA

**Keywords:** biosimilars, cell therapy, FDA, gene therapy, intellectual property, manufacturing

## Abstract

This article provides an early analysis of the potential for creating future biosimilar competition for cell and gene therapies (CGTs) to lower prices and improve patient access, building on a unique set of interviews with relevant experts. Our discussion addressed regulatory, manufacturing, intellectual property, and market size challenges. Due to CGTs' complexity, meeting the regulatory requirement of ‘high similarity with no clinically meaningful differences’ will be difficult. Gene therapies are likely better candidates for biosimilar development than cell therapies. Biosimilarity should be met when gene therapy biosimilars contain the same genetic sequence as a reference product, and the variability in the vector meets the high similarity standard. Manufacturing challenges, including the lack of standardized platforms, high production costs, and complexity, pose significant obstacles. It may also be important to demonstrate biosimilarity within the manufacturing process. Intellectual property barriers, specifically patenting, trade secrecy, and regulatory exclusivity, could hinder biosimilars' ability to gain market share, although recent Supreme Court decisions limiting the breadth of patent claims could ease barriers to future CGT competition, including from biosimilars. Finally, inadequate market sizes might create hurdles, especially for curative treatments, as patient pools shrink following treatment by the reference CGT.

## I. INTRODUCTION

Revolutionary cell and gene therapies (CGTs) have recently begun to provide new treatment options for patients with previously untreatable conditions and are poised to disrupt the treatment paradigm for genetic disorders, cancers, and other chronic conditions by delaying disease progression or providing cures. However, they have also created significant challenges due to their high prices, with the potential to significantly increase spending and stretch payers’ budgets.

Although key underlying technologies often originate from the National Institutes of Health (NIH) or other publicly funded research institutions,^1^ payers across all sectors of the health care system face economic challenges when covering and paying for CGTs. Not only do CGTs have high upfront costs but it may be difficult to predict how many individuals in a patient pool will need the CGT^2^ because the population of patients eligible within a payer’s group may fluctuate and could significantly change from one period to another. These issues are a particular problem for states, as they are required to maintain balanced budgets and set Medicaid managed care organization capitation rates annually, and for small businesses with limited reserves because even a single case could lead to substantial cost implications. Payers also express concerns regarding the evidentiary uncertainty about the long-term effectiveness and durability of CGTs at the time of approval, and the risk of loss of return on investment.^3^ While these evidentiary challenges may be shared with other high-cost specialty drugs, CGTs typically require a one-time upfront payment for a single administration of the treatment, a feature that poses a risk to budgets and cash flows, especially for states that must set their budgets in advance for one- to two-year periods by legislative vote, and which often cannot run annual deficits.^4^ Furthermore, the multi-payer healthcare system in the US allows patients to change insurance plans, and payers may be more reluctant to pay the expected value of a CGT upfront, since a subsequent insurance plan may reap the benefits later on.^5^ Patients are also affected by these challenges, as they limit the ease of access to treatment and patient decision-making due to, eg cost-sharing and formulary management tools.

Currently approved CGTs and many now in late-stage development are for rare diseases or indications with small population sizes. Given the number of therapies in development and the high list prices of all CGTs to date, CGTs are expected to have a significant impact on health system budgets even if they individually treat a relatively small patient population. Going forward, moreover, CGTs may not be limited to small patient populations. Due to further scientific advances, CGTs are increasingly being developed for more common diseases.^6^ Additionally, costly forms of cell therapies known as chimeric antigen receptor T-cell therapies (CAR-T therapies) are expanding indications and patient populations.^7^ The trend toward more prevalent diseases will exacerbate the budget impact of CGTs. Importantly, however, as we discuss in detail below, larger markets may also contribute to more favorable conditions for competition by biosimilars or branded competition.

Developers may follow alternative paths for competitor products. They may generate their own trial data and file a Biologic License Application for a new CGT within the same therapeutic class that is different enough from the first product that it does not infringe the first product’s patents. Alternatively, developers might wait until the loss of exclusivity of an innovator product, and produce a highly similar product that draws from innovator development and manufacturing practices using the so-called biosimilar pathway, which we describe in Section III. Because we are concerned about cost and access, and the introduction of biosimilars may be a crucial strategy for reducing cost and improving access, this paper focuses on the biosimilar option. This strategy is particularly important for CGTs, given their high associated costs and groundbreaking potential in patient care. However, CGTs are very complex biologics that are likely to pose significant challenges to competition through the development and approval of biosimilars. Moreover, because CGTs are so new, there has thus far been little scholarly discussion of the legal, scientific, and economic terrain facing potential CGT biosimilars. This article comprehensively surveys this terrain.

## II. METHODOLOGY

We integrate literature from multiple disciplines and also utilize a unique set of interviews with 21 subject matter experts to gather broad perspectives on four key areas (regulatory, manufacturing, intellectual property, and pricing/payment) that future biosimilar candidates will need to navigate to enter the field. Our subject matter experts include seven regulatory experts, five manufacturing experts, four intellectual property (IP) legal experts, and five pricing and payment experts. In addition to their subject-matter expertise, many of these experts also represent key stakeholder types that will be crucial to biosimilar CGTs’ success including regulators from the Food and Drug Administration (FDA), commercial payers, drug manufacturers, IP attorneys who specialize in pharmaceuticals, manufacturing organizations, and several academic experts who enriched our insights with their understanding and experience primarily in the manufacturing, IP and payment spaces. Finally, our experts also include former government personnel from pivotal agencies in this space, such as FDA and the Centers for Medicare and Medicaid Services (CMS). These experts were chosen through a mix of purposive and snowballing sampling approaches, initially based on publicly available information from our review of peer-reviewed and grey literature, our past work on biosimilar- and CGT-related topics, and recommendations from experts identified in our initial research.

This article addresses sequentially the challenges of FDA regulation (Part III), manufacturing (Part IV), intellectual property (Part V), and pricing and payment (Part VI). Throughout we present not only challenges but also opportunities for overcoming challenges.

## III. REGULATORY CONSIDERATIONS

### III.A. Current Regulatory Challenges in the Novel CGT Space

The FDA regulates the approval of CGTs and also regulates the approval of biosimilars. Predicting the regulatory environment for potential CGT biosimilars requires an understanding of both the current regulatory environment for originator CGTs as well as for biosimilar products, and of how the two regulatory domains are likely to intersect. Indeed, follow-on CGT product development is conceptually strained by the uncertainties still present in the regulatory environment for novel CGTs, and therefore enhancing regulatory clarity for novel CGTs will help shed light on how biosimilarity will be characterized for these products.

CGTs represent multiple classes of transformational treatments to mitigate disease progression and return or increase functionality with the potential to achieve durable or prolonged effectiveness, potentially making them a preferred treatment option.^8^ Yet, the regulatory environment for CGTs, which harness increasingly complex technologies,^9^ is only in its infancy. Throughout the development lifecycle, CGT manufacturers face challenges such as assuring quality through manufacturing and testing as well as efficiently demonstrating safety,^10^ efficacy, and durability.^11^

These hurdles, alongside the need for more FDA guidance and research on novel CGTs, as our background research and discussions with experts have identified, underscore the complexity of bringing CGT products to market.

Many of the experts we interviewed called for further guidance documents on relevant topics like manufacturing, testing, donor materials, and stability of constructs for CGTs. One expert pointed out that past biosimilar studies for even less complex therapeutic protein products have revealed reference product inconsistencies. Further regulatory guidance, including information regarding opportunities for flexibility, will allow sponsors to fully understand CGT reference products before biosimilar development, which will ultimately inform standards for biosimilar characterization and development in this space. This is especially true for cell therapies given the complexity of living cells. FDA has existing high-level guidance on preclinical research considerations and recommendations specific to CGTs.^12^ In addition, the agency has highlighted safety concerns and the lack of clinical experience with CGTs for sponsors to consider when designing early-phase clinical trials of CGTs.^13^ These guidance documents represent a starting point to establish standards for CGTs, with more clarity needed for complete characterization and clinical development.

CGT clinical trials are plagued by holds, accounting for approximately 61 percent of all holds issued by FDA’s Center for Biologics Evaluation and Research (CBER) in 2022,^14^ (while trending downward in absolute numbers, from a high of 147 in 2018 to 70 in 2022). Congressional leadership for the House Energy and Commerce Committee’s Health Subcommittee prioritized the topic of clinical holds for CGTs in a letter to the CBER director and in a subsequent hearing.^15^ The issue of clinical holds comes as research in CGTs has skyrocketed to 1687 clinical trials according to figures from a recent industry report.^16^ Often, clinical trial holds are lifted yet some companies choose not to continue with trials.^17^

To address the backlog of CGT trial holds, FDA has released a draft guidance to encourage sponsors to create a single ‘umbrella’ trial to study multiple versions of CGT for a single disease^18^ and reorganized the Office of Tissues and Advanced Therapies into the Office of Therapeutics (OTP), with the purpose of explicitly expediting the review of CGTs.

### III.B. Biosimilar Regulatory Classification

The biosimilar regulatory pathway derives from the Biologics Price Competition and Innovation Act (BPCIA) passed as part of the Patient Protection and Affordable Care Act.^19^ Biosimilar products are shuttled through an abbreviated 351 k approval pathway designed to reduce development time and cost while maintaining safety and effectiveness safeguards. The goal of this process is not a standalone approval—as with the 351a pathway—but rather to ensure that the safety, effectiveness, and purity of the biosimilar are comparable to the reference product. Biosimilar products must demonstrate that they are ‘highly similar to’, and possess ‘no clinically meaningful differences’ from, the reference product.^20^ The biosimilar designation is distinct from interchangeability, which, if granted by FDA, and subject to applicable state law, means that a pharmacist can substitute the biosimilar for the reference product without seeking the provider’s authorization. Most CGT administration occurs in complicated hospital settings, rendering interchangeability a minor concern^21^ (although we note that perceptions around the interchangeability designation may still hinder uptake and substitution, even if administered outside the pharmacy setting).^22^

The biosimilar development and approval process typically requires a comprehensive package primarily consisting of analytical data, notably structural and functional characterization.^23^ In the context of therapeutic proteins, FDA has issued numerous guidance documents for informing biosimilar development, including scientific considerations for demonstrating biosimilarity.^24^ The higher degree of flexibility inherent to the abbreviated pathway makes for less emphasis on conducting large-scale comparative clinical trial data to demonstrate safety and efficacy as compared to reference products.^25^

Generally, animal studies are only run if there are additional safety concerns with the biosimilar that need to be resolved before initiating clinical trials. Appropriate clinical pharmacokinetic (PK) and pharmacodynamic testing is expected, as is at least one clinical comparative immunogenicity study between the proposed biosimilar and reference product. ^26^

Two key concepts in biosimilar approval inform what evidence is needed—the ‘stepwise approach’ and ‘totality of evidence’.^27^ The ‘stepwise approach’ describes the notion that each next step towards approval is focused on addressing unanswered regulatory questions from previous steps. Each successive step targets ‘residual uncertainties’ until they are all resolved. ‘Totality of evidence’ refers to the idea that biosimilarity with the reference product is confirmed through the collective assessment of various measures (analytical, preclinical, and clinical) and each measure alone isn’t enough to confirm biosimilarity.

Currently, given that the novel CGT development environment is still in its infancy, the regulatory pathway for biosimilar products has yet to interact with the regulatory pathway for CGTs. The current biosimilar pathway and statute have been interpreted with therapeutic protein products in mind. As the two paths begin to intersect—with the potential future development of biosimilars for CGTs—stakeholders will need to address a number of regulatory considerations. Understanding what the FDA considers to be ‘highly similar’ with ‘no clinically meaningful differences’ for CGTs will be key.

### III.C. Defining Biosimilarity for CGTs

Experts suggested factors that will help address the definition of biosimilarity—namely the primary mode of action for the product. The primary mode of action for the CGT directly relates to the quantity and complexity of its functional elements. Cell therapies such as CAR-T work by removing a patient’s cells, modifying them with a standard vector and selected gene of interest, processing the modified cells, and finally inserting them back into the patient. They differ from gene therapies consisting of standardized vectors and selected genes of interest that are directly administered to patients. The more elaborate modes of action for CGTs present more challenges for defining biosimilarity.

### III.D. Gene Therapy Biosimilar-Specific Considerations

Because their functional elements are different, different CGT products present unique considerations for examining potential biosimilarity. Gene therapies are defined by genetic sequencing corresponding to introduced genetic material. The nature of the introduced genetic material is usually in the form of a transgene, a nucleic acid sequence encoding for the gene of therapeutic interest.^28^ The vehicle for delivering the introduced genetic material into the cell, the vector, is typically derived from viruses (adeno-associated viral, adenoviral, lentiviral, or retroviral).^29^ The delivery mechanism for the gene therapy product is impacted by the choice of vector.

The consensus among our interviewee experts was that gene therapies may lend themselves to future biosimilar development and are more feasible to make into biosimilars than their cell therapy counterparts. Because the major functional element of the product will be the transgene itself, one can establish the identity of what is being produced in the follow-on product, and its biosimilarity to the reference product, by examining the genetic sequence. A gene therapy that has a similar construction for transgene expression, containing the same desired genetic sequence as a reference product, may reasonably fit the concept of biosimilarity. The therapy’s delivery vehicle, ie any differences in vectors, would also have to be considered for showing high similarity—even if they deliver the same gene of interest. However, some experts theorized that some flexibility in vector variability will be permissible from a regulatory perspective. The exact differences in the vectors will need to be well characterized for any functional or analytical differences. Given the centrality of manufacturing to the therapy itself, even if the viral vector is the same, it will still be important to demonstrate biosimilarity within the manufacturing process using purity markers such as the level of capsids filled and DNA contamination. Experts also noted that clinical trials for comparative immunogenicity are more easily performed with gene therapies than for cell therapies. The exact immunogenicity considerations for gene therapy clinical trials would still need to be defined. Experts also noted that durability will most likely be a concern for gene therapy biosimilars. A better understanding of mechanisms of action, off-target functions, differences in vectors, and other aspects that could impact genetic alteration would be critical, likely necessitating the development of new technologies.

### III.E. Cell Therapy Biosimilar-Specific Considerations

Beyond considerations for the genetic material and vector in the case of genetically modified cell therapies, added complexity for cell therapies includes the functional elements of the cellular starting material and final product formulation, potentially rendering these products very difficult to ‘biosimilarize’. The cellular starting material can be derived from either the patient’s own body, in an autologous treatment, or from a donor or donated umbilical cord blood, in an allogeneic treatment.^30^ The final product formulation for cell therapy is either infused shortly after formation into the patient or cryopreserved for later infusion.^31^ Analytical and functional characterizations for cell therapies, needed to ascertain biosimilarity, would need to be carefully designed so as to not destroy the final cell-based product. Clinical trials for cell therapy biosimilars would likely involve more steps than trials for gene therapy biosimilars to discern differences in immunogenicity.

To the extent cell therapy biosimilars were at all possible, experts had differing opinions on which cell therapies might lend themselves best to proving biosimilarity. Some hypothesized that genetically modified autologous cell therapies such as CAR-T could be subject to biosimilarization if the biosimilar developer could demonstrate that their vector was identical or highly similar and resulted in no clinically meaningful differences compared to the reference cell therapy’s vector. Additional evidence for biosimilarity would have to also come from the yet-to-be-defined critical quality attributes leading to cell characterization after the transduction of the starting cell material with the vectors.

Other experts believe, however, that the self-donor nature of these products makes them inherently unsuited as an economic matter to the regulatory biosimilarity paradigm. These experts contended that allogeneic ‘off the shelf’ products that a larger patient population can take will better lend themselves to biosimilarization, in part because many doses can be manufactured in reproducible campaigns. However, identifying the reference product will be difficult because the starting cellular material is so vast, despite well-standardized procedures for harvesting, and differences in the source of the donor material as well as its heterogeneity could make it challenging to demonstrate the same level of efficacy, requiring additional scientific methodologies to enable this process. The starting genetically modified cell materials used in allogeneic products would need to meet a high standard of consistency.

There remains the possibility that certain cell therapy products are suitable for biosimilarity, while for others biosimilarity is not feasible. The aforementioned viewpoints contrast the qualifications for autologous versus allogeneic cellular therapies. As further technological progress is made and foundational knowledge is gained to define the critical quality attributes for cellular therapies, the applicability of the current biosimilar paradigm to these therapies will become clearer.

In addition, cell lines shift in characteristics over time, complicating the already complex quality parameters needed for the establishment of biosimilarity. These products will need to demonstrate consistency in manufacturing, and developers will need standard practices for harvesting donor cells. As described in more detail below, achieving standardization in manufacturing will be burdensome for developers and may require navigating legal and resource barriers.

### III.F. Challenges for CGT Biosimilar Development

For sponsors considering a development program for CGT biosimilars, there are several challenges present through the process leading up to and including regulatory review. The potential for biosimilar development for CGTs may depend on how well these challenges are understood by sponsors and regulators. In order to foster competition within this future environment, alignment will be needed on evidence submission standards, manufacturing processes and validation, and regulatory review.

### III.G. Evidence Submission Standards

Evidence standards for CGT biosimilars will encompass both the product specifics and the production process. Experts expressed a need to define the correct critical quality attributes and parameters for analytical comparability exercises in order to establish biosimilarity. A similar process unfolded for therapeutic proteins as scientific research permitted advanced characterization of protein structure and function, which led to FDA guidance on demonstrating comparability.^32^ Experts suggested a similar scientific process would take shape for CGTs that may lead to eventual guidance on comparability assessments for CGT biosimilars. Manufacturing and process standards will also need to align including the harvesting method, how cells are expanded and transfected, and cell characteristics as transfected cells are formulated. In terms of evidence collection, demonstrating proof of concept and long-term safety and efficacy of products will be the most important consideration for CGT biosimilars. Experts also expressed concern about demonstrating durability and emphasized a need for long-term data collection to understand mechanisms of action and safety.

While animal studies are less prioritized in studies for therapeutic protein biosimilars, they may play a large role in CGT biosimilars, which raise many unanswered questions and safety concerns, most notably about the permanency of the genetic alteration created by gene therapy. There is also a larger, ongoing effort by FDA to advance alternative methods for pre-clinical testing.^33^ From a proof-of-concept standpoint, leveraging new technologies for in vitro and in silico testing—in addition to the refined use of animal models—will be needed for the assessment of comparability for CGT biosimilars. However, in human clinical studies, biosimilars will face the same barriers as innovator CGTs since these products cannot be tested in healthy subjects, and exposure responses vary from traditional medical products. Some clinical data will be needed, but will most likely come from small clinical trials that will not be the primary focus of developers. However, our experts did voice doubt over the feasibility to conduct these trials when reference product data is available. The unique nature of CGTs also poses ethical considerations for using a control group in advanced clinical testing. Because many CGTs target rare and life-threatening conditions for which no effective treatment exists, it could be unethical to withhold a potential therapy from patients in need. Moreover, as some CGTs are tailored to a patient’s particular genetic makeup, the creation of a standard control group becomes challenging. Furthermore, clinical trials for CGT products present unique questions given the possibility of one-time treatment with a durable effect.

### III.H. Regulatory Review and the Distribution of Expertise within FDA

In contrast to therapeutic biologics, which are generally reviewed by the Center for Drug Evaluation and Research (CDER), CGTs are reviewed by multiple divisions within the newly formed Office of Therapeutics within the Center for Biologics Evaluation and Research (CBER). In addition, CBER has established an interdisciplinary center team to promote engagement with prospective innovators and developers and sponsors regarding advanced manufacturing technologies, the CBER Advanced Technologies Team (CATT).^34^ For potential CGT biosimilar sponsors that use advanced manufacturing technologies, coordination between CATT and review divisions within CBER can help address perceived regulatory barriers that may otherwise prevent manufacturers from adopting advanced manufacturing technologies and ultimately entry into the CGT biosimilar market.

Additionally, the CDER at FDA has wide-ranging expertise in both rare disease and biosimilar research and review. CDER’s Accelerating Rare Disease Cures (ARC) Program is scoped to speed the development of rare disease treatments and coordinate all work that could affect rare disease development, such as novel clinical trial designs and endpoints and stakeholder outreach.^35^ ARC will ‘address challenges with well-established trial designs, endpoint selection with a limited understanding of the natural history of the disease, and give advice on performing and interpreting rare disease clinical trials with small patient populations’.^36^ It serves as ‘connective tissue’ throughout the agency rather than a discrete center of excellence, to spread expertise.^37^ Biosimilar expertise at FDA is spread across CDER, including within the Office of Therapeutic Biologics and Biosimilars, the Office of Biotechnology Products, and the Office of Clinical Pharmacology. Typically, the review of biosimilar products is coordinated with the same review division for reference products. CGT biosimilar reviews will likely present a need for coordination across CBER reference product review divisions within OTP and CDER staff experienced with rare diseases and biosimilar research.

### III.I. Future Regulatory Outlook

#### 1. Legal and regulatory compatibility of follow-on CGTs with the existing biosimilar pathway

As our experts noted, the statutory definition of biosimilarity may not need to be modified to accommodate CGT biosimilars due to the flexible nature of the existing biosimilar pathway. The regulatory standards for and scientific framework around biosimilarity will need to evolve as more is learned about novel CGTs. Experts discussed the introduction of new programs to potentially introduce modified standards for establishing CGT biosimilarity. They also emphasized that FDA still needs to address guidance questions around protein biosimilars, particularly as more innovative biosimilars come to market and more issues arise from the application of regulations that were written with older biologics in mind around approval and post-approval processes to newer biosimilar approvals. New guidance for CGT biosimilars can be considered in parallel with additional guidance for therapeutic protein biosimilars.

#### 2. Actions by FDA

FDA’s Biosimilars Action Plan intends to standardize the review of biosimilars, enhance the Purple Book, explore data-sharing agreements with foreign regulators, publish guidance on labeling, and provide additional support for developers on considerations of product quality and questions about the manufacturing process.^38^ Similarly, FDA’s Research Roadmap for Biosimilars seeks to enhance biosimilar development with specific priorities for regulatory impact focused on increasing the accuracy and capability of structural and functional characterizations as well as alternatives to comparative clinical studies.^39^

Stakeholders agreed that any pathway or initiative for CGT biosimilar approval will require clear communication and frequent meetings with sponsors so that they understand the feasibility of their product and sufficient guidance. However, stakeholders emphasized that FDA does not currently have the capacity to process all the CGT applications received, and struggles to produce timely guidance. The increased OTP staff designed to respond to CGT development under PDUFA VII may provide the needed resources for current CGT approval and future BsUFA reauthorizations may include staffing for future CGT biosimilar development.

The 2023 Consolidated Appropriations Act directed FDA to create a platform technology designation for drugs or biologics that use technologies already found in an approved product.^40^ This is an agency-wide initiative aiming to increase efficiencies in drug development, manufacturing, and regulatory review. The director of OTP has indicated that a platform approach may benefit gene therapies.^41^ FDA is due to release draft guidance on the platform designation program. While the recommendations for this program are still forthcoming at the time of writing this paper, the platform designation may prove to be an incentive for sponsors to pursue gene therapy development.

## IV. MANUFACTURING CONSIDERATIONS FOR CGT PRODUCT BIOSIMILARS

CGT manufacturing is a complex and expensive process. Experts report that CGT manufacturing was initially characterized by underfunding and low production volumes, largely due to a limited number of approved products and small patient populations. However, in the last 5 years, investment in CGT development and manufacturing has increased, although CGT manufacturing infrastructure is still catching up to growing production demands, which only continue to increase.

Challenges in CGT manufacturing persist, which will impact potential biosimilar development. Process complexity, combined with limited volumes and the need for significant manufacturing expertise, contributes to a very high cost of goods sold (COGS). While not necessarily representative of COGS for many CGTs, one industry leader estimated that gene therapy COGS can range as high as $1 million, excluding any other development costs.^42^

Lack of platform standardization across products likely contributes to high costs. Unlike other drug product types, where ‘plug-and-play’ platforms are available for common manufacturing steps,^43^ costly, bespoke manufacturing processes are often developed for new CGTs due to their production complexity. This lack of process standardization may hamper the ability of biosimilar developers to create competing products that can meet biosimilarity thresholds. CGT process standardization could encompass the processes and equipment used for critical stages such as hosting, processing, and purifying viral vectors, as well as cell collection, isolation, transfection, culturing, and washing. Furthermore, standardizing quality checks could enhance both the reliability and scalability of CGT production, facilitating a more streamlined pathway from development to delivery. Further investment in CGT development also needs to be encouraged to create a robust market that provides reasonable expectations for return on investment. Addressing these issues in the current CGT landscape can increase developer entry and innovation in the space while facilitating eventual CGT biosimilar entry.

### IV.A. Overview of the Current CGT Manufacturing Field

#### 1. The Manufacturing Process

As previously mentioned, CGT manufacturing is complex and requires significant expertise. Because of its intricacy, the manufacturing process is an essential part of the product. The process also differs greatly between gene and cell products. In gene therapies, which typically utilize viral vectors, the genetic sequence of interest can be substituted into the viral vector. The viral vector also encodes for proteins responsible for creating the protein shell of a virus, called a capsid, as well as other proteins needed for replication. For the assembly of certain vector types, eg, adenovirus and adeno-associated viral vectors, additional materials including an inert, helper virus or shuttle vector can be used to create the engineered viral vector. Manufacturers then use certain cell types to host the vector and allow it to reproduce so that there is a large enough quantity to harvest. In the downstream process, the viral vector is processed and purified to remove non-essential components and then packaged and sent away for use.^44^^,^^45^ Once injected into the patient, the vector binds to the cell membrane and travels through the cell where it injects its genetic sequence into the nucleus.^46^

In cell therapy, such as CAR-T, T-cells are collected through leukapheresis, cryopreserved, and then sent to the manufacturing site. There, they are isolated, and viral DNA that codes for receptors that bind to antigens on the surface of cancer cells are transfected into the cells which now better recognize cancer cells. These cells are multiplied in culture, washed, and undergo a thorough quality check before being shipped back to the patient (or multiple patients, in the case of allogeneic therapies) for injection**.**^47^

#### 2. Manufacturing Costs Estimates for CGTs

Experts highlighted the high labor costs in manufacturing CGTs, requiring many specialized roles with a range of levels of education and training. The lack of qualified workers with adequate specialization in training has contributed to high labor costs and high turnover in manufacturing roles. Lower-level positions like line operators may require at least an associate’s degree and prior training. Once hired, these workers will still require training to develop competency, including understanding complex testing and assay processes. More skilled workers like quality control and assurance employees need an advanced degree and a high level of technical training. Recruiting and retaining highly skilled employees requires large investments, which may be particularly challenging for smaller and new companies.

When examining gene therapies specifically, labor costs represent a large portion of COGs. One analysis found that labor and indirect costs represented 35 and 40% of costs, respectively. The other top contributors to COGS include raw materials and quality-related activities. The production of viral vectors utilizing labor and raw material resources is a key driver of cost—representing between 15 and 50% of COGS with the currently available manufacturing technologies.^48^ Significantly, however, some research suggests that the uptake of more platform technologies like single-use bioreactors can save up to 90% of COGS per dose, in large part due to the savings from the technology’s scalability in moving towards industrial manufacturing levels and lowering associated labor and indirect costs.^49^

Across estimates for the cost of cell therapy manufacturing, labor is consistently the highest cost factor.^50^ The development and manufacturing COGS for cell therapies may also vary by product. In the case of CAR-T manufacturing, developers may have significantly lower COGS when deriving multiple therapeutic doses from a single manufactured batch for allogeneic products as opposed to retaining only one therapeutic dose per manufacturing process for autologous products.^51^

#### 3. Key Actors

CGT manufacturing principally occurs through academic medical systems with the appropriate resources, Contract Development and Manufacturing Organization (CDMOs), and internal manufacturer infrastructure.^52^ Small biotechnology companies and academic medical systems led the way in initial CGT development, while larger and more established companies took a back seat. As of February 2020, only 15% of launched CGTs originated from or were owned by a top 20 biopharmaceutical company.^53^ While this percentage has increased somewhat since 2020 due to approvals of products from Kite Pharma, Celgene, and Janssen, a majority of CGTs still do not originate or are owned by a top 20 biopharmaceutical company. However, most large biopharmaceutical companies now have CGTs in their pipeline, and about half of the 20 largest have expanded their CGT portfolio through acquisitions. Simultaneously, academic medical systems have moved beyond research to contribute to manufacturing capacity, including the development of manufacturing sites to speed up production.^54^

CDMOs also play an important role in CGT manufacturing, as they offer production resources that may be critical for nascent developers. Experts agreed that contracting with CDMOs is most advantageous early in manufacturing when developers aim to rapidly move through the process but lack the resources to do so. For newer companies that have difficulty setting up and maintaining their own manufacturing capabilities, CDMOs offer the ability to easily start and stop manufacturing as needed through development phases. However, as the development process moves along, typically by phase 3, developers tend to expand their in-house capacities for manufacturing and develop an internal, bespoke process without CDMO involvement. Although they are important to the development process, CDMOs have a limited capacity for CGT manufacturing—experts report that most CDMOs are working to expand their abilities to serve the variety of CGT developers.

#### 4. Fostering a Competitive Market for CGTs

Facilitating biosimilar CGT market access should center on addressing persistent challenges to manufacturing, including related problems of lack of standardized platforms to guide manufacturers in best practices; the COGS of production, particularly for new or smaller companies; the complexity of the manufacturing process itself; and the lack of an appropriately skilled manufacturing workforce.

Of course, some currently ascendant firms may have an interest in maintaining bespoke practices, complexity, and tight control over their workforce. Idiosyncratic, complex, and tightly guarded manufacturing practices could enhance prospects for intellectual property (specifically patents and trade secrets) that creates barriers to entry. We discuss intellectual property issues in Part V. That said, even for ascendant firms, standardization and reduction in complexity could reduce their own COGS, and this reduction should provide a countervailing incentive.

### IV.B. Standardization and Reducing the Cost of Production

The high cost and complexity of CGT manufacturing pose a barrier to the development of both innovator CGTs and potential biosimilar products. Addressing the current challenges to CGT production is necessary in order to encourage production from a spectrum of developers with varying resources, and to foster the appropriate environment for biosimilar development.

### IV.C. Increasing Standardization

Experts agreed that one of the best ways to foster entry into the CGT market is by promoting the adoption of platform standardization for manufacturing and testing processes. Platform standardization will also lower the cost of production as manufacturers can leverage economies of scale, more accurately pinpoint the most cost-effective raw materials and labor necessary prior to production, and reduce time to production. With the eventual development of CGT biosimilars, standardization should also help address questions about proving biosimilarity in the manufacturing process.

Reaching widespread standardization faces several challenges. Barriers include CGT developers’ strong regimes of secrecy surrounding their bespoke manufacturing processes, secrecy that is often maintained through reliance on trade secret law and insufficient disclosure in manufacturing process patents. The relative nascence of CGT manufacturing may also pose questions about best practices for developing platforms. Developing these platforms across companies will also require Good Manufacturing Practices (GMP) oversight and prior knowledge of platform utilization that companies may not have.

The nature of the product and its variability, particularly for cell therapy products, poses a further problem for achieving standardization. For example, it is common for the processes used to characterize viral vectors to be developed from the ground up for each therapy or manufacturer, which leads to higher costs and variation in vectors from one product to the next. An approach that could help facilitate standardization is a regulatory process for viral vector characterization that is akin to drug master files. Specifically, agreement on standardized quality attributes and vector development methods could lead to FDA ‘pre-approval’ for development that could drive down costs, enhance the control strategies for CGT products, and promote additional product development. Potency testing will also be important to analytical methods used to characterize cell therapies, but potency relies on functional assays that are difficult to standardize for living cell products. One expert predicts that process standardization challenges will eventually be addressed in part by engineering out immunogenicity measures using allogeneic cells and pairing these measures with more advanced analytical methods to set a product test that will prove standardization.

The FDA Advanced Manufacturing Technologies Designation Program that was created by the Consolidated Appropriations Act of 2023 enables expedited regulatory review of drugs and biologics that are manufactured utilizing designated advanced manufacturing technologies. This program could provide an incentive to manufacturers to use common manufacturing platforms across multiple CGTs in order to gain expedited review timelines.

Standard coordinating bodies will play a key role in developing and facilitating the use of standardized technical approaches. National coordinating bodies are developing standards for specific products or procedures. The United States Pharmacopeia (USP) is developing new standards for elements of CGT manufacturing, as well as utilizing best practice guidance in existing USP publications.^55^ Further USP standards will be incorporated into FDA regulation, which will play a large role in encouraging standardization across the industry. USP standards could also provide necessary transparency to CGT biosimilar developers when the time comes to replicate innovator manufacturing processes. Given the nascence of CGT development, enforcing standards too quickly may stifle the ability of innovators to develop promising yet complex products. When enforcing manufacturing platform standardization, USP, FDA, and other standards setters will need to find a careful balance between fostering innovation in new manufacturing processes and enabling biosimilar development. Over time, priorities should shift towards enabling biosimilar development.

The Standards Coordinating Body (SCB) is also at the forefront of convening and coordinating stakeholders to develop standards for CGT manufacturing processes. While their work is not incorporated into the regulatory process, they receive funding from FDA to coordinate community efforts for standards development, which may facilitate the incorporation of standards with widespread stakeholder support into regulatory guidelines.^56^ Experts report that SCB may benefit from increased outreach to industry partners to encourage cross-collaboration, particularly as support for standardization across the CGT space grows with increased product development.

International bodies can also play a role in fostering community cooperation and manufacturing standardization. International accreditation bodies like the Foundation for Accreditation of Cellular Therapy can encourage widespread standardization with the breadth of their oversight. While developing international standards is time-consuming and does not legally bind companies to best practices, standards can foster shared expectations between national regulatory authorities^57^ and help authorities work together to address bottlenecks or challenges to standardized platform use. Other organizations like the International Society for Cell and Gene Therapy can foster consensus among stakeholders on best practices for commercialization and workforce training to further inform industry standardization efforts.

### IV.D. Reducing Cost of Production

Addressing production costs is important for novel CGT developers to foster product development and streamline the production process, and for potential CGT biosimilar developers for whom low production costs will make market entry more feasible. New approaches to reducing the cost of production utilize innovations in research and development, engineering quality control, supply chain, and other technical expertise. Two possible approaches to reducing costs are scale-up and automation (potential methods of and considerations for these approaches are detailed below.)

#### 1. Scale-Up

Scaling-up CGT production, for example through producing larger batch sizes or higher volumes, can lower COGS and the price per therapeutic dose, ultimately allowing more manufacturers to enter the current CGT market and facilitate biosimilar development. This suggestion focuses on the advantages of allogeneic cell therapies or gene therapies, where larger batch production can lead to significant cost savings. Even for autologous therapies, certain standardized processes, like quality checks, could be scaled to apply across multiple patient treatments. This approach would maintain the personalized aspect while exploring efficiencies in the manufacturing process. There are several potential approaches to scaling-up CGT production. While centralized manufacturing is the more traditional method and can lower manufacturing costs through economies of scale, decentralized manufacturing may also present opportunities for scaling up CGT manufacturing. Decentralized manufacturing can increase development capacity and more rapidly identify and address consumer needs. However, decentralized manufacturing has its own complexities, and may entail fewer economies of scale, due to the need for distributed manufacturing expertise, expenses associated with distribution of raw materials, higher automation requirements between sites, and the need for partnerships between sites to share pre-competitive information.^58^

In some cases, starting with small-scale production for new cell therapy technologies before moving to commercial-scale manufacturing may initially be advantageous since innovations can be implemented more quickly and tests are less cost-prohibitive.^59^ Developers can also take advantage of the small-scale solutions that already exist for the cell therapy manufacturing process downstream from cell expansion.^60^ Commercial-scale manufacturing however may require rethinking the production processes that were used in the early stages of development in order to realize lower scale-up costs.^61^

There are different considerations and opportunities for scale-up within CGT products. In gene therapy production, viral vector technology has the potential to play a large role in scale-up. Viral vector production capacity has been limited by the increasing number of therapies in development and growing target populations for treatment.^62^ This has resulted in a viral vector shortage, with estimates that the current vector capacity can cover only 5–10% of the manufacturing capacity needed in the next ten years.^63^ However, the development of new viral vector production methods can facilitate increased manufacturing capacity and scale-up. More vector manufacturing facilities with increased production volume capacity are in development—although these come with their own challenges for production at a large scale.^64^ Like cell therapy products, developers should also consider their choice of viral vector. Certain vectors are cheaper, or have less lot-to-lot variability. For example, lentiviral vectors may be better suited to scale-up than gamma retroviral vectors and non-viral methods because they can be directly added to cell culture vessels.^65^

Scale-up methods for cell therapies must also consider the kind of cells used, as some cell types are easier to manufacture and subsequently scale up than others.^66^ There are multiple technologies for cell development that provide opportunities for scale-up through their various features.^67^

#### 2. Automation

Automating manufacturing to use less labor-intensive and less variable processes provides a solution to the time and labor costs of CGT production. Reducing variability may also reduce difficulties in demonstrating biosimilarity. One 2018 estimate found that the use of automation in manufacturing an autologous T-cell product could save up to 72% of the labor costs.^68^ Another 2018 study highlighted a partly automated process as the most favorable scenario for the future of CGT manufacturing—this would require less labor without costly automation systems that may require further streamlining to increase batch production.^69^ Closed, automated processes also provide a GMP environment with automated hardware that is equivalent to or better than highly trained workers in GMP facilities.^70^

There are currently first-generation automated platforms on the CGT production market that integrate several manufacturing steps into one machine. However, second-generation platforms that are largely in development may fully automate production and eliminate the need for manual labor at any point of production.^71^ One platform already on the market integrates cell activation, transduction, application and final harvesting in one unit. Another product still in development has the potential to automate tissue collection, isolation, cell expansion, harvesting, concentration, and cryopreservation.^72^ These platforms could significantly reduce the need for skilled laborers and lower barriers to entry for newer CGT developers looking to manufacture products efficiently and at low cost. In addition to developing enhanced automation platforms, addressing challenges with automated processes can facilitate platform use and entry into manufacturing. Within automation, developing more process analytic technologies (PAT) can move forward the use of feedback-driven improvements. Stakeholders at a 2018 workshop identified bioprocess analytics, for example on more complex real-time culture data measures, as the largest technical challenge to the adoption of automation in CGT manufacturing.^73^

### IV.E. Reducing Complexity in Manufacturing Processes

Stakeholders can harness certain manufacturing techniques to encourage participation and foster the CGT market in preparation for biosimilar entry. Centralized manufacturing could benefit from lessons learned through the blood supply chain model utilized by blood banks. Areas for analogous insights include the high-speed and high-volume management of procurement in the blood supply chain, administration with track and trace capacity, and segregation of lots with embedded quality control.^74^

The more nascent decentralized manufacturing technique can potentially enhance current manufacturing processes. Through a hub and spoke model, manufacturing could be established at centers of excellence that work under the supervision of a central hub site. Each facility would be connected through cloud-based systems that allow for the implementation of the same manufacturing protocols, in-process and batch release assays, and quality attributes.^75^ This model allows for the recruitment of skilled workers with fewer geographic constraints, but requires high coordination between sites and could potentially dilute manufacturing expertise.^76^

Similarly, point-of-care manufacturing offers the opportunity for manufacturing in various areas, and at the patient’s site of clinical care. However, this requires building GMP facilities in a hospital setting, with experienced staff to run facilities and implement quality control and standardization efforts.^77^ If stakeholders are able to build and operate these facilities, point-of-care manufacturing could eliminate some parts of the manufacturing process, like the cryopreservation of materials for distribution.^78^ Manufacturers may also better respond to clinical demand when planning production. This manufacturing approach, and the methods described above, provide alternative processes that may bolster developer interest in the current CGT market and facilitate biosimilar entry.

### IV.F. Knowledge Transfer

Ensuring an adequately sized and trained workforce is necessary for the successful production of CGTs. Experts highlighted the need for skilled employees with knowledge of CGT production but acknowledged the challenge that the nascence of the CGT space poses. This will be particularly true for CGT biosimilar manufacturing, as skilled workers may not be aware of or interested in employment opportunities in the very early stages of the field. Based on past experiences with biosimilar development, knowledge transfer may play a critical role in developing a skilled workforce.

Experts indicated that legacy biosimilar companies acquired crucial information on manufacturing practices from knowledge transfer as experts moved around different companies. CDMOs also house the necessary manufacturing know-how that smaller generic and biosimilar developers can utilize through manufacturing contracts. However, given the complexity of CGT manufacturing and commonly bespoke processes, knowledge transfer may be more challenging in CGT biosimilar development. Experts indicated that standardization efforts can play a large role in advancing knowledge transfer as this will encourage or require developers to move away from bespoke processes. Within companies, data extrapolation between product versions can also speed up knowledge transfer, which will be supported by further FDA guidance on their use of extrapolation between CGT products.^79^ Finally, patents and trade secret protection of manufacturing processes need to be addressed. We discuss these issues in the next section.

## V. INTELLECTUAL PROPERTY CONSIDERATIONS FOR FUTURE CELL AND GENE THERAPY BIOSIMILARS

### V.A. Overview

Therapeutic protein biologics are typically covered by many more patents than small molecule drugs. ^80^^,^^81^^,^^82^ We do not possess the full number of biologic patents as manufacturers are not required to list them with the FDA unless they are involved in litigation, in contrast to the small molecule drugs’ ‘Orange Book’. However, a recent study found that between 2010–2023, 271 patents associated with 12 originator biologics were involved in litigation.^83^ Various authors attributed these ‘thickets’ of patents in part to the complexities associated with producing and using biologic drugs, including their formulation, analysis, and manufacturing.^84^

Manufacturing process patents also appear to represent a particularly effective way of blocking competition from biosimilars. In fact, one study found that almost 50% of the patent assertions against biosimilars involve manufacturing process patents.^85^ Additionally, these patents are typically sought later in the product exclusivity lifecycle, often for strategic reasons, as what is filed later will also expire later.^86^ Indeed, because the way in which a biologic is manufactured affects its safety and efficacy, it is sometimes said that for biologics, ‘the process is the product’.^87^ This is even more true for CGTs, where there is no clear compound and the treatment constitutes a fundamentally more complex process than traditional drugs. Given the centrality of the manufacturing process to CGT, experts noted that the manufacturing process patent landscape is significantly more substantial for CGT than for traditional biologics, allowing originators to claim manufacturing aspects such as the vector and its various constructs, cell line preparation, the transfection process and analytics and quality control.

Because of the complexity of biologic manufacturing, manufacturing-related patents are not the only form of IP blocking biosimilars from entering the market. Critical manufacturing techniques and know-how can also be protected as trade secrets, a form of IP that differs from patents in that it is not time-limited (as long as the brand manufacturer can keep it a secret). Additionally, unlike with patents, competitors can still use the protected information if they independently discover it.^88^ Patents and trade secrets are often used together to protect the manufacturing processes of biologic inventions. Additionally, because patent disclosure obligations are not necessarily enforced rigorously by the USPTO, they can cover the same information. To some extent, both forms of IP serve the same purpose—excluding competitors from using the invention. However, the details of the protection offered by the two regimes differ substantially: while patent law requires adequate disclosure in exchange for a 20-year period of exclusivity, and also protects against independent invention, trade secrecy requires no disclosure and confers exclusivity until the secret information is independently invented or reverse engineered. Thus, while manufacturers may seek patents on any parts of the manufacturing process that will be discovered relatively quickly by competitors in any event, manufacturers may prefer to use trade secrecy to protect aspects that are unlikely to be independently discovered or reverse-engineered by competitors. For these latter aspects, trade secrets may provide protection from competition longer than the 20 years provided by patents. As with patents, experts have noted that they witness more trade secrets being used in the CGT space than for traditional biologics, due to the many different steps that are required to create these complex products.

As mentioned above, even for those aspects of the manufacturing process they do cover, patent claims may fail to disclose sufficient information. In the absence of critical knowledge protected by trade secrecy, it may be time-consuming for a competitor to produce a therapeutically equivalent biosimilar, resulting in long periods of no competition.^89^ IP protections can also be stacked to maximize the duration of protection, for example, by first relying on trade secrecy rather than patents to keep information secret, and then filing for patents later in the life cycle of the product, when a would-be competitor may have gotten closer to reverse-engineering the secret, thereby, extending the years of protection and delaying biosimilar entry.^90^ Although standard patent law doctrine barring patents on information that is already being used commercially should prevent this type of strategic behavior, it is not clear that the USPTO enforces these standard legal requirements.^91^

### V.B. Current CGT Patent Landscape and Relevance for Future Competition

Many patent applications are filed on CGTs. For example, an analysis of just gene therapy patents in 2021 reported about 2000 patents filed per year from 2000 to 2020 with the US accounting for over half of the world’s total gene therapy patents.^92^ Additionally, in 2019, there were 683 CAR-T patent publications including 124 granted patents in the US.^93^ The patent landscape is likely to become increasingly crowded and complex, especially as patents are relatively inexpensive to obtain.^94^ In the case of large firms, US filing fees for patent applications are higher, but they remain easily affordable. Experts have noted that for CGT, there is no single product that is protected by patents, as is the case for other pharmaceutical products, but that there is robust IP around the protection of the complex process that is associated with the products. For example, the process of making gene therapy involves the making of a virus, the culturing of the cell to produce the virus, and providing the virus to the patient, thereby creating multiple steps prior to getting to the ‘product’ itself. At every step, IP can be created, and a potential biosimilar competitor will need to address this IP in order to enter the market.

### V.C. The Breadth of CGT Patent Claims

In addition to the sheer numbers of CGT manufacturing process patents, the breadth of these claims is relevant to biosimilar competition. The broader a patent claim, the more difficult it is for a would-be biosimilar entrant to use alternative processes that don’t infringe the claim. In a testimonial to the importance of patents, the question of CGT patent breadth has already been litigated. The breadth issue came up in the *Kite v. Juno* case, where Juno Therapeutics sought to show that Kite was infringing Juno’s CAR construct patent (Patent ‘190).^95^ Juno claimed the CAR’s binding element to target proteins cancer cells, known as the scFv (single chain variable domain), according to its function of being capable of binding to these targets (ie broad claiming), as opposed to claiming the scFv according to its specific, narrower structural features.

Yescarta was covered by the ‘190 Patent claim because it contained an scFv capable of binding to a particular target, CD19, found on certain cancer cells. Juno sued Kite, alleging that Kite’s scFv (targeting CD19) infringed the ‘190 patent. In response, Kite defended that the ‘190 Patent claim was overly broad and invalid for failing to meet the so-called ‘written description requirement’,^96^ which requires the inventor to describe the invention that it seeks to patent,^97^ reflecting one aspect of the disclosure requirement in the patent system described earlier. Meeting this legal standard usually involves disclosing a number of examples, typically by structure, that are supposed to be representative of the full scope of the patent claim.

The California district court ruled in favor of Juno, finding that it met the written description requirement and that Kite infringed the ‘190 Patent.^98^ The Federal Circuit later reversed, ruling that Juno’s patent disclosure was insufficient in that it did not cover any scFv binding element capable of interacting with any selected clinical target, failing to demonstrate that it possessed the full scope of the claimed invention at the time of filing.^99^ To put the point more simply, the Federal Circuit held that Juno’s claims were overly broad, as they attempted to capture much more than what Juno had actually invented and disclosed.

Although Juno then appealed to the Supreme Court, the Court opted not to take that particular case. Thus, the Federal Circuit’s decision represents the most relevant current law with respect to CGT claims specifically.

Additionally, the overall reasoning of the Federal Circuit in *Juno v. Kite* has been bolstered by the Supreme Court in another case, *Amgen v. Sanofi,* addressing very similar issues for ‘ordinary’ antibodies. In *Amgen*, the Court ruled in a manner that directly supports the Federal Circuit’s analysis of CGT patent breadth in *Juno v. Kite*. Thus, we turn next to *Amgen v. Sanofi*.

Amgen had appealed to the Supreme Court with a case involving a disclosure requirement closely related to the written description known as enablement.^100^ Amgen appealed after the Federal Circuit decided that two Amgen patents covering all antibodies claimed by their function of binding and blocking a key site on the PCSK9 molecule, were invalid.^101^

In May 2023, the Court unanimously affirmed the invalidation, agreeing with the Federal Circuit that the claims were overly broad. It appears that for now, the enablement and written description disclosure standard for patent claims will continue to put broad functional claims in pharmaceutical companies’ patents at risk of invalidation. As our experts noted, these cases suggest that CGT manufacturers may need to become more comfortable with narrower patent claims, protecting their own space instead of attempting to seize the product space more broadly, as stringent legal standards may only permit developers to claim the product by structure, eg amino acid sequence, rather than a broad claim on function. For example, in the CAR-T space, this development may imply that companies would need to specifically define the structure of the CAR that binds to the cancer cells.

We note that these cases address not potential patent infringement by a biosimilar competitor but instead by another originator product. Nevertheless, these legal developments can also be expected to have implications for future competition from biosimilars, limiting the breadth of patent claims for a CGT product developer seeking to block biosimilar entry. The existing case law may benefit biosimilars because it likely means that for now, an originator CGT cannot dominate the whole field, potentially providing some room for biosimilar ‘design arounds’ that do not infringe reference product patents, so long as they are viewed by FDA as still meeting the standard for high similarity with no clinically meaningful differences from the reference product. But as we have also noted, CGT products involve a substantial amount of IP, including a multitude of trade secrets covering the process, so even if they have to address a narrower set of patent claims, potential competitors may still find the IP environment extremely challenging to navigate. Even when entrants can work around existing patents, questions will remain on what knowledge needs to be gleaned elsewhere, and how other IP protections like trade secrets will impact the know-how necessary to make the product.

### V.D. Regulatory Exclusivities

Whether or not claiming CGT products broadly turns out to be challenging for the innovator, regulatory exclusivity may be a critical component of the protection of these therapies. In particular, there are two types of regulatory exclusivities provided by FDA that are applicable in the CGT space: the 12-year biologic data exclusivity that, during its term, prohibits reliance by the biosimilar on originator clinical data and the 7-year orphan drug exclusivity applicable if the CGT meets the Orphan Drug Act definition for a drug that it is intended for the treatment of a rare disease or condition. (So far, all approved gene therapies address orphan diseases^102^.) Both of these exclusivities could be important tools in possibly delaying the entry of a would-be biosimilar.

While in many cases thus far, biologic patents have expired after the termination of the 12-year biologic exclusivity (highlighting the extent of biologic drugs’ patent arsenals),^103^ some experts still emphasized the utility of this exclusivity for the originator. This may be because some of the later-filed patents that extend exclusivity past the 12 years are more vulnerable to invalidity challenges.

The second type of exclusivity, the orphan drug exclusivity, has been highlighted as particularly important as well while raising specific questions and considerations for CGTs. Generally speaking, the orphan drug exclusivity prohibits FDA from approving the ‘same drug’ for the same use or indication for 7 years after the date of approval^104^ unless the second drug is shown to be clinically superior.^105^ Therefore, determining a drug’s ‘sameness’ is critical for the enforcement of the orphan drug exclusivity and consequently, a potential biosimilar’s timing for entering the market. More specifically, if FDA deems a potential biosimilar sufficiently ‘different’ that it falls outside the originator’s orphan drug exclusivity but nonetheless sufficiently the ‘same’ for purposes of FDA approval under the BPCIA, then the orphan drug exclusivity is substantially weakened. In contrast, if the ‘sameness’ standards for the Orphan Drug Act and the BPCIA are interpreted in a strictly parallel fashion, then the stakes for competitive entry do not change. Because the competitive stakes could be high, we discuss this intricate issue in some detail.

For biologics, FDA defines ‘same drug’ as a drug that contains the same ‘principal molecular structural features (but not necessarily all of the same structural features), and is intended for the same use or indication as a previously approved drug, except that, if the subsequent drug can be shown to be clinically superior, it will not be considered to be the same drug’.^106^ However, current regulations provide no specific definition for gene therapies. In 2021, FDA issued final guidance^107^ describing its interpretation of how the regulatory ‘sameness’ criteria apply to gene therapies in the context of the Orphan Drug Regulations. The guidance stipulated that assuming that they are intended for the same use or indication, two gene therapies that express different transgenes or use entirely different vectors are different drugs for purposes of the orphan drug program because they will not contain the same principal molecular structural features.

In the context of gene therapies, the key puzzle for ‘sameness’ under the Orphan Drug Act involves how different a vector can be. FDA clarified that two gene therapy products that use vectors from different viral groups will be considered different. Additionally, vectors from the same viral group (eg AAV2 vs. AAV5, or gamma retrovirus vs. lentivirus) may be considered different when the differences between them impact factors such as tropism, immune response avoidance, or potential insertional mutagenesis. Finally, FDA intends to determine whether variants of a vector from the same viral group (eg AAV2 vs. a variant of AAV2) are the same or different on a case-by-case basis. The FDA is unlikely to consider polymorphisms or other minor differences as leading to a new drug, however, the ‘minor differences’ and ‘additional features’ language may need clarification.

Thus, under this guidance, a product that used the same transgene but a slightly different vector or vector variant might in some cases be able to enter the market despite the Orphan Drug Act exclusivity. For purposes of potential biosimilar competition, the key question would then be whether the product was ‘highly similar’ under the BPCIA.

While ‘highly similar’ is not necessarily the same standard as ‘same’, subject matter experts see a close relationship, noting that the approach taken by the FDA under the Orphan Drug could easily funnel into biosimilar requirements under the BPCIA. Of course, if the requirements are interpreted in an entirely parallel fashion, then the competitive stakes for biosimilars do not change.

We note that another aspect of the breadth of orphan drug exclusivity has been recently litigated in court. In *Catalyst Pharms., Inc. v. Becerra*, the Court of Appeals for the 11th Circuit held that the Orphan Drug Act confers exclusivity on the entire treatment area for a product even if the product is only approved for certain indications of use.^108^ FDA recently announced it would comply with the decision as applied to that case but would continue in all other contexts to apply its existing regulations that tie the scope of exclusivity to approved indications. Nevertheless, this 11^th^ Circuit decision may have a chilling effect on follow-on developers.

Finally, we note that because the orphan drug exclusivity’s length is 7 years, it will likely be superseded by the longer 12-year biologic exclusivity, diminishing its value to the originator product in protecting against biosimilar entry, unless it is provided for a second indication after the 12-year exclusivity has expired. Under this scenario, the non-orphan indication can be carved out from the label,^109^ but access might still be blocked for the orphan indication.

In sum, a large array of intellectual property protections – patents, trade secrecy, and regulatory exclusivities create barriers to entry for CGT biosimilars. Of these, trade secrecy may be most challenging given its indefinite length.^110^ That said, it bears mention that under blackletter patent law, if an originator uses a manufacturing process in secret, another firm (including a biosimilar firm) that independently invents the process could patent it^111^ and assert the patent to block the originator’s prior secret use.

## VI. PRICING AND PAYMENT CONSIDERATIONS

### VI.A. Anticipated CGT Competition Dynamics

Addressing pricing and payment barriers to CGT biosimilar adoption will be crucial to the success of the biosimilar market. This section explores some of the barriers faced by biosimilars generally and how these barriers may apply to CGT biosimilars in particular if or when they are available.

### VI.B. Market Share and Market Size Barriers and Incentives

Experts broadly agreed that the shrinking market size for biosimilar CGTs referencing curative originator therapies would be a considerable deterrent for follow-on manufacturers. If the innovative CGT is curative, the patient population (which, in many cases, is already small for CGTs) will be reduced over time to only the prevalence of new cases of the disease. In some disease areas, such as cystic fibrosis, the prevalence of new patients is so low that there probably will not be a market for follow-on competition, and many CGT markets will be too small to justify even branded competitors if the first manufacturer is ahead of them by many years. However, whether the patient pool would actually shrink remains unclear because we do not yet have definitive evidence about the durability of ‘curative’ CGTs, at least for certain genetic subsets of a given patient population.

Inadequate market size may be a barrier for other entrants even if the patient population does not shrink for low-prevalence diseases as manufacturers may not see sufficient incentives to enter the market with a biosimilar since they would be splitting the market with the originator. Moreover, as experience in the small molecule space shows, prices only dramatically decline with the entry of at least three generic competitors.^112^ This level of entry may be difficult to achieve when there is a small patient population, further limiting the potential for substantial price reductions.

However, incentives for market entry could be present for CGTs that address larger populations and/or non-curative CGTs. Potential examples include a sickle cell disease CGT in the pipeline, therapies that target macular degeneration, hemophilia (eg the recently approved Roctavian for adults with severe hemophilia A^113^), and certain cancer CGTs. While CGTs are not expected to be patient-administered, and thus the FDA interchangeability designation will not be relevant for purposes of substitution by retail pharmacists, perceptions around the designation may still matter somewhat; Uptake may be hindered if providers view CGT biosimilars as equivalent and interchangeable to the originator product only if they obtain an interchangeability designation.^114^ Recently, however, FDA reversed its stance on disclosing interchangeability in the biosimilar’s label,^115^ which could suggest a shift in the agency’s approach to reducing the importance of this designation. Additionally, CGT biosimilars may not face the same switching-related barriers faced by biosimilars for maintenance and treatment of chronic diseases because of their one-time administration, thus mitigating barriers to switching. Messaging to providers and payers about the similarity of CGT biosimilars, particularly in terms of safety and durability, will be important to support their acceptance of these complex products.

Rebate walls are another threat to potential CGT biosimilar market share and incentives to launch, especially for commercial insurers.^116^ During the period of several years between now and when CGT biosimilars become feasible, if they do, the FTC may act to address rebate walls (as well as patent thickets) and other related issues.

Finally, experts noted that there will be branded competition for some disease areas, especially those with larger patient populations, making it important to consider to what extent the newer, competing therapy is better than the established therapy. When there is branded competition with sequential innovation, patient ‘warehousing’ could take place. That is, patients may not be treated with the available established therapy, for a clinical trial of the new therapy, or for treatment with the new therapy once it is approved. This phenomenon would have an impact on market incentives for the launch of a competing product if it is able to demonstrate clinical improvements compared to its predecessor. It could reduce the likelihood that there will be a biosimilar entrant for the first product if the would-be biosimilar manufacturer knows there will be such sequential innovation and patient warehousing.

### VI.C. Particular Considerations for Future CGT Competition by Payer Type

#### 1. Medicare

Experts familiar with Medicare noted that they generally do not expect unique payment-related barriers or disincentives in Medicare for potential future CGT biosimilars compared to traditional biosimilars. The discussion below details particular considerations for each payment program within Medicare.

#### 2. Medicare Part A

Many of the CGTs currently on the market and in the pipeline are covered in the Medicare inpatient Part A benefit, which pays for care based on predetermined diagnosis related group (DRGs) for the inpatient stay and applicable treatment. Biosimilars are bundled like other drug products into the applicable DRG under Medicare Part A, and drugs may be entitled to a new technology add-on payment designation, which means that hospitals are eligible for a temporary additional payment outside of the DRG.^117^ The capitated payments to providers in Part A may create incentives for providers to use lower-cost drugs and treatments, such as lower-cost biosimilars when they are available and/or negotiate for lower drug prices when there are alternative treatment options to give the providers leverage for negotiation.^118^ Because of this, Part A providers may be more likely to use lower-cost generic or biosimilar drugs, with potentially promising implications for the prescription and uptake of lower-cost CGT biosimilars in the future—especially future CAR-T biosimilars and competitor products in other therapy areas that may be provided in inpatient care settings.

While the uptake of biosimilars for CAR-T could be facilitated by the existence of an applicable DRG, biosimilar price differences compared to their reference product might not be big enough to shift market share in Part A because list prices may not differ significantly between CGT originators and biosimilars. Data on biosimilar drug utilization rates in Part A is limited because (1) existing biologic biosimilars are predominantly covered by Part B and (2) there is little available evidence breaking down drug spending under Part A. Reporting such prescription drug use is not required, and CMS has struggled with this lack of utilization data; Payers have noted that they generally have little insight into spending on inpatient drugs.

#### 3. Medicare Part B

Medicare Part B is important for the CGT payment landscape because the earliest-approved CGTs, CAR-T therapies, are likely to be increasingly administered to patients in the outpatient care setting covered by Part B. Approved gene therapies, and other products in the pipeline are also, or will likely be, covered by Part B. More broadly, most ‘traditional’ biologics and biosimilars are covered under the Part B benefit. Biosimilars have demonstrated mixed results in terms of their ability to penetrate the healthcare market and generate meaningful cost savings and changes in the list price of a given reference biologic in response to biosimilar entry have also varied.

Subject to the particular considerations associated with the development and entry of biosimilar CGTs, there may be informative examples of the impact of biosimilars for biologic drugs in Part B that can shed light on the possible future impact of competition in CGTs.^119^

Biosimilars are reimbursed at their own average sales price (ASP) but receive the same add-on payment as the reference biologic to limit provider financial incentives to pick the higher-cost originator product. While the equal add-on payment aims to ensure that providers are not encouraged to utilize the more expensive originator biologic, it does not sufficiently encourage providers to switch to the biosimilar. Further, the use of unique billing and payment codes and reimbursement rates means that the differences in ASP do not translate into a difference in net provider reimbursement, so price competition between the biologic reference products and their biosimilars is dampened. Thus, Part B payment policy reform that increases price competition between biosimilars and their reference biologics has been proposed to decrease total drug spending.^120^

The 2022 Inflation Reduction Act (IRA) includes a five-year temporary increase to the Part B add-on payment for certain biosimilars to incentivize their use, increasing the payment from 6% to 8% of the reference product’s ASP for biosimilars introduced between 2022 and 2027.^121^ The increased add-on payment could meaningfully improve the uptake of biosimilars in Part B. But biosimilars for CGTs are not expected to be introduced before 2027, so it is unlikely this payment policy change in the IRA will directly affect CGT biosimilar uptake unless the applicable period for the add-on payment increase is extended beyond 2027.

Payment for ancillary services is also needed for CGTs. While the 6% add-on payment for Part B drugs (or 8% in the case of some biosimilars under the IRA) could compensate for some ancillary services, ancillary costs could still pose a challenge in CGT payment structures. Patient-centered payment models for CGTs could help address ancillary costs of treatment (eg travel and follow-up care), integrate service delivery with the cost of therapy, and help with the collection of postmarket data. Shifting away from fee-for-service (FFS) while incorporating a condition-specific payment approach in such a model may incentivize the use of lower-cost biosimilars if available.

In FFS Part B, beneficiaries with no supplemental insurance—about 16% of the Medicare population in 2019^122^— are subject to a 20% coinsurance with no out-of-pocket (OOP) maximum. This is not the case in Medicare Advantage (MA) (and other commercial plans) where most patients with conditions that require CGTs are likely to reach their OOP maximum whether using the originator or the biosimilar, so OOP will not serve as an effective cost lever for CGTs and their potential biosimilars.

### VI.D. IRA Drug Price Negotiations in Part B

The IRA also established the Medicare Drug Price Negotiation Program that authorizes Medicare to negotiate drug prices for certain high expenditure, single source drugs covered by Medicare Part B or Part D; the first-year Part B drugs will be subject to price negotiation is 2028 when 15 Part B or Part D drugs will be chosen by CMS.^123^ Experts offered their opinions on the potential for the IRA drug price negotiation provisions to impact CGTs in the future. Some believe that CGTs are not likely to meet the required thresholds for IRA drug price negotiation, which are based not on a price per unit but on total expenditures, unless there are new products for much bigger patient populations resulting in substantial expenditures to the program, for example, a CGT for age-related eye disease. Others highlighted that the insufficient patient population sizes after the period of 11 years—when the biologic will be eligible for negotiations and most patients have likely already been treated—would render these treatments irrelevant for price negotiations. The impact of the IRA price negotiations on CGT biologics will also be limited if the majority of CGTs remain in Part A, however, stakeholders and background literature suggest that more products will be administered in the outpatient setting in the future, eg with CAR-T shifting to outpatient administration. ^124^ CMS confirmed in its revised guidance^125^ on the drug price negotiations for the first year of the program that CGTs are not categorically ineligible for the plasma-derived product exclusion from negotiations, and whether a CGT therapy qualifies for the exclusion will be assessed using the same standards as other biological products. CMS intends to refer to the FDA-Approved Blood Products website in determining whether a product is derived from human whole blood or plasma and consult with FDA as needed.^126^ The orphan exception could also exempt CGT products from the negotiation program if the criteria are met—the product must be approved for an indication (or indications) for a single rare disease or condition. A CGT’s selection for negotiation under the IRA could have downstream impacts on the development of potential biosimilars because the lack of predictability could deter investments by biosimilar manufacturers and lead to even longer-term originator company monopolies.^127^ Conversely, the threat of IRA price negotiation could make it so that a manufacturer of an originator product prefers to allow a selected competitor into the market to avoid negotiation.^128^

### VI.E. Medicaid

Medicaid programs and managed care organizations (MCOs) may contract with a pharmacy benefit manager (PBM) to negotiate supplementary rebates and implement their own maximum allowable cost lists for multi-source drugs, with potential implications for CGT competition. In Medicaid, biosimilars are treated like originators and must pay brand-level statutory rebates including inflation-based rebates. The current discount structure in Medicaid may not be set up to incentivize biosimilar uptake. Research has suggested that state Medicaid agencies that administer their drug benefit using FFS (compared to MCOs) and that operate preferred drug lists (PDLs) tend to prefer originator drugs over biosimilars because originators with higher list prices that are accompanied by what are often very substantial rebates, sometimes provide lower net costs than biosimilars.^129^ Moreover, the large price increases that often occur with branded drugs may actually provide an incentive for states to prefer these products over their follow-on versions due to the inflationary rebate component that can reach 100% of the drug’s average manufacturer price.^130^ The American Rescue Plan enacted a proposal to eliminate the Medicaid drug rebate cap and the policy will go into effect in 2024.^131^ Lifting the rebate cap might further provide lower net costs for branded drugs over generics and biosimilars.^132^

Many experts expressed particular concerns about the ability of Medicaid programs to handle the costs of CGTs indicated for children, and those for conditions with larger patient populations like Sickle Cell Disease (SCD), proposing potential approaches to help Medicaid including having the federal government guarantee access and provide underwriting to ensure Medicaid patients will get access to CGTs (even before biosimilars could become available). With previous biosimilar experience demonstrating that the price difference between the originator and biosimilar has not always been as big as was hoped,^133^ there is a worry that any CGT biosimilars are likely to still have very high launch prices so the more general concerns about Medicaid’s ability to pay for CGTs will continue to be relevant even after competition enters. In an attempt to address this challenge, some states ask for reporting from manufacturers if a biosimilar is priced at less than a 15% differential from the reference product.^134^

Outcomes-based contracts (OBCs) may be helpful for CGTs with limited evidence of durability, with rebates based on insufficient performance or lack of durability. The Biden administration’s executive order encouraging the Center for Medicare and Medicaid Innovation to look into alternative payment models for drugs^135^ could lead to new possibilities for outcomes-based contracts in Medicaid. This led to the February 2023 HHS announcement on drug prices that includes a CGT access model for Medicaid.^136^ The Cell and Gene Therapy Access Model, which is still being developed, would enable Medicaid agencies to assign CMS to coordinate and administer multi-state OBCs.^137^

Currently in Medicaid, CGTs are often separated from existing payment bundles because they are too new and the state wants to ensure access to these needed therapies for its Medicaid patients. Stakeholders noted that the product may sometimes be donated by the company or heavily discounted, including through clinical trial use. Still, Medicaid pays for the time the patient is in the hospital to manage the immunological response (often in the ICU) and other costs associated with the use of the CGT; even without the cost of the CGT itself, these are very expensive treatments for Medicaid to cover, an issue that may still pose a challenge with the introduction of biosimilars.^138^

### VI.F. Commercial Payers

The outlook for CGT competition in the context of commercial payers may be similar to traditional biologic-biosimilar competition, in which dynamics related to PBMs and formularies play a significant role in the market. The potential role and implications of rebate walls, hospital markups, reinsurance, and care delivery organizations (including infusion centers and specialty pharmacies) for CGT therapy pricing, payment, and competition in the commercial insurance market were discussed with experts.

Manufacturers of the originator CGT may use rebate walls and other strategies leveraging their market dominance to maintain a preferred formulary placement and other advantages with a suppressive effect on competition. The current state of payment and reimbursement for outpatient biosimilars covered in commercial markets is complicated because hospitals are typically reimbursed on a ‘percent-of-charge’ basis—a discounted rate off a price that is multiple times the Wholesale Acquisition Cost. The incentives in this reimbursement model may lead providers to favor the administration of the higher-cost originators over biosimilars.

There is limited transparency on hospital markups in the drug distribution chain. One approach to combat markups is ‘white bagging’, wherein insurance companies cover medications distributed by a specialty pharmacy instead of allowing providers to conduct ‘buy-and-bill’. As payers would like to move away from buy-and-bill, they would generally welcome a potential direct payment mechanism for CGT biosimilars, while manufacturers may be reluctant to change their distribution and payment position. Most large commercial insurers entered the specialty pharmacy market to control costs of relatively more complex, higher-cost treatments and then created specialty pharmacy group purchasing organizations to have more leverage to buy at a larger scale and further reduce costs. Nevertheless, specialty pharmacies currently dispensing CGTs and other intermediaries are paid as a percentage of list price, and control where the volume of sales go, and thus may not have incentives to switch to lower-cost biosimilars when available.

More insurers are also buying infusion centers or companies to control the costs of infusion and infusion drugs. To support this move toward infusion centers, payers will want FDA policy and CMS policies that allow for CGT infusion in care settings outside of hospitals. At the same time, health systems consolidating around the infusion of CGTs will be able to impose prices on payers because of their greater negotiating power, continuing to encourage the use of buy-and-bill and having implications for the incentives for continued use of higher-priced CGTs. MA plans are expected to try to control the costs and utilization of CGTs to keep their premiums lower, possibly through the use of restrictive networks.

### VI.G. Pricing and Payment Approaches for CGTs During the Post-Exclusivity Phase, Including if CGTs cannot be Biosimilarized

If CGTs cannot be made into biosimilars, experts expressed hope for branded competition in the post-exclusivity phase, especially for conditions with larger patient populations. For example, there are at least three products in development in the SCD space^139^^,^^140^ each with slightly different indications to carve out their own market share although over time there may be off-label use and indications may drift. Such brand-to-brand competition and/or ‘creative destruction’ through sequential innovation is likely to tame the prices of CGTs, possibly including through rebates or discounts related to the uncertainty of long-term outcomes. In brand-to-brand competition, the first entrant will set a market price and branded competition could facilitate downward price pressure through competition and/or incremental improvements. There could be a role for the FDA to encourage branded competition through, eg expedited pathways or other levers to incentivize and prioritize more CGT applications. The FDA could create an action plan to promote branded competition for high-cost therapies similar to its existing biosimilar action plan.

For a host of reasons, some stakeholders were skeptical about the feasibility of the government regulating prices for CGTs through contractual genericization, ie mandatory price reductions that would provide manufacturers with a certain, reasonable margin above the cost of production if there is no follow-on entry for these products after a long period of exclusivity. They suggested the government could instead take a potential role in CGT procurement through centralized purchasing with negotiation, similar to the approach it used during the peak of the COVID-19 pandemic for vaccines and treatments. Centralized purchasing can involve transparency and public input, encouraging manufacturer participation. In contrast, others believe that CGTs that have higher prices out of sync with estimates such as those created by ICER should face legislation or regulation (including HTA application) in several years due to the challenges in sustaining those prices in the long term, especially given the current landscape and momentum in drug pricing policymaking.

In the scenario where CGTs cannot be biosimilarized and aside from potential brand-to-brand competition, some experts emphasized the importance of alternative payment mechanisms for these therapies and finding ways to lower their prices. Such models, specifically payment over-time approaches and OBCs may be useful to pay for CGTs that cannot be made into biosimilars. OBCs are particularly useful when there is uncertainty about the durability of the treatment and payment in those contracts could also be linked to non-health outcomes such as a reduction in total costs of care and cost offsets, which may encourage the use of a biosimilar (if available) or lower-cost branded competitor.

However, some stakeholders have raised their concerns about the usefulness of OBCs for CGTs because of challenges in collecting the necessary data and addressing patient churn. Many Medicaid agencies do not have the capacity to negotiate individual agreements for the CGTs coming on the market or track the outcomes needed for OBCs.^141^

Large, even national risk pools can help pay for CGTs. Cigna’s Embarc has taken a market-based approach to control CGT prices by creating as big a risk pool as possible to spread the expense of the catastrophic event (needing CGT) over enough people to make it financially sustainable. This reinsurance mechanism helps shield health plans and customers from the financial risk of high-cost gene therapies and combines health services, medical management, and specialty pharmacy expertise.^142^ Cigna planned to expand the drugs included in Embarc, including CAR-T therapies and additional gene therapies as they are launched.^143^ This approach of spreading the expense in a financially sustainable way will become more challenging when the population of eligible patients for CGTs gets larger as more therapies with bigger patient populations are expected to come on the market in the coming years.

## VII. CONCLUSION

CGTs present potentially transformative opportunities for patients but pose challenges for payers due to their high costs. Taking steps today to enable and encourage future biosimilar competition in these markets can support greater patient access and affordability for payers. Drawn from a unique set of interviews with a range of relevant experts, as well as an extensive literature review, this paper has outlined key regulatory, manufacturing, IP, and pricing/payment challenges, as well as mechanisms that could help overcome these challenges. Key areas for engagement to facilitate future CGT competition include encouraging regulators to provide greater clarity and explore process standardization requirements for CGTs. Additional research into the market potential and likely IP barriers for CGT competition (both branded and biosimilar competition) is also important for promoting a competitive landscape in the future.

## Footnotes

1 Kerstin N. Vokinger, Jerry Avorn and Aaron S. Kesselheim, ‘*Sources of Innovation in Gene Therapies — Approaches to Achieving Affordable Prices,’*  New England J. Med. (2023).

2
Gregory W. Daniel et al., Breakthroughs and Barriers: Advancing Value-Based Payment for Transformative Therapies, Duke-Margolis, May 2019, https://healthpolicy.duke.edu/sites/default/files/2021-05/advancing_vbp_for_transformative_therapies.pdf; MACPAC, *Addressing High-Cost Specialty Drugs*, Report to Congress on Medicaid and CHIP, June 2021, https://www.macpac.gov/wp-content/uploads/2021/06/Chapter-1-Addressing-High-Cost-Specialty-Drugs.pdf.

3
*Id.*

4
Gregory W. Daniel et al., *supra* note 2.

5
Gregory Daniel et al., *Advancing Gene Therapies and Curative Health Care Through Value-Based Payment Reform*, Health Aff. Forefront, October 30, 2017, https://www.healthaffairs.org/do/10.1377/forefront.20171027.83602/full/.

6
*Regenerative Medicine: Disrupting the Status Quo*, Alliance for Regenerative Medicine Annual Report, 2021, http://alliancerm.org/wp-content/uploads/2022/03/ARM_AR2021_FINAL-singles.pdf.

7
*Piping Hot: A Look at the State of Cell, Gene and RNA Therapies in Early 2023*, IQVIA blog, May 2, 2023, https://www.iqvia.com/blogs/2023/04/piping-hot-a-look-at-the-state-of-cell-gene-and-rna-therapies-in-early-2023.

8
Sandeep R. P. Kumar, David M. Markusic, Moanaro Biswas, Katherine A High and Roland W. Herzog, *Clinical Development of Gene Therapy: Results and Lessons from Recent Successes*, 3 Mol. Ther. Methods Clin. Dev. 16034. May 25, 2016

9 Maria Aspioti and Paolo Siciliano, *CAR-T & Beyond: CGTs in Development in 2022*, Cell Gene, https://www.cellandgene.com/doc/car-t-beyond-cgts-in-development-in-0001 (2022).

10 Halioua-Haubold, Celine-Lea et al., *Regulatory Considerations for Gene Therapy Products in the US, EU, and Japan*, 90 Yale J. B. Med.,683–693. December 19, 2017.

11 FDA, *Long Term Follow-Up After Administration of Human Gene Therapy Products, Guidance for Industry,* January 2020, https://www.fda.gov/media/113768/download.

12 Center for Biologics Evaluation and Research, *Preclinical Assessment of Investigational Cellular and Gene Therapy Products*, U.S. Food and Drug Administration (2019), https://www.fda.gov/regulatory-information/search-fda-guidance-documents/preclinical-assessment-investigational-cellular-and-gene-therapy-products (last visited Jul. 24, 2022).

13 Center for Biologics Evaluation and Research, *Considerations for the Design of Early-Phase Clinical Trials of Cellular and Gene Therapy Products,* US Food and Drug Administration (FDA, Feb. 10, 2020), https://www.fda.gov/regulatory-information/search-fda-guidance-documents/considerations-design-early-phase-clinical-trials-cellular-and-gene-therapy-products.

14
Brenda Sandburg, Gene And Cell Therapy Clinical Holds Decline While Those For Drugs Hit 12-Year High, Pink Sheet (2023), http://pink.citeline.com/PS148691/Gene-And-Cell-Therapy-Clinical-Holds-Decline-While-Those-For-Drugs-Hit-12-Year-High?utm_source=search&utm_medium=RSS&utm_term=&utm_campaign=search_rss (last visited Sept. 27, 2023).

15 Guthrie, *Eshoo Raise Concerns on FDA Backlog of Cell and Gene Therapy Applications*, U.S. Congressman Brett Guthrie (2023), https://guthrie.house.gov/news/documentsingle.aspx?DocumentID=387916 (last visited Sept. 27, 2023).

16
Sector Snapshot, (2023), http://alliancerm.org/wp-content/uploads/2023/08/20230808-Sector-Snapshot_Final_2023.pdf.

17 Max Bayer, *Legend Scraps Early-Stage CAR-T for Lymphoma 6 Weeks after Clinical Hold Lifted*, Fierce Biotech (2022), https://www.fiercebiotech.com/biotech/legend-scraps-early-stage-car-t-lymphoma-six-weeks-after-clinical-hold-lifted (last visited Jul. 24, 2022).

18 Center for Biologics Evaluation and Research, *Studying Multiple Versions of a Cellular or Gene Therapy Product in an Early-Phase Clinical Trial*, U.S. Food and Drug Administration (2021), https://www.fda.gov/regulatory-information/search-fda-guidance-documents/studying-multiple-versions-cellular-or-gene-therapy-product-early-phase-clinical-trial (last visited Jul. 24, 2022).

19 Charles B. Rangel, *H.R.3590 - 111th Congress (2009–2010): Patient Protection and Affordable Care Act*, (2010), https://www.congress.gov/bill/111th-congress/house-bill/3590 (last visited Aug. 2, 2021).

20 US Food and Drug Administration, *Biosimilars Info Sheet: Biosimilar Regulatory Approval Pathway*, https://www.fda.gov/media/154914/download#:∼:text=Therefore%2C%20all%20biosimilar%20and%20interchangeable,result%20from%20the%20manufacturing%20process (accessed Sept. 2023).

21 Center for Drug Evaluation and Research, *Biosimilar and Interchangeable Products*, FDA (2019), https://www.fda.gov/drugs/biosimilars/biosimilar-and-interchangeable-products (last visited Jul. 24, 2022).

22 M. D. Allan Gibofsky, M. D. Colby Evans & M. D. Vibeke Strand, *Provider and Patient Knowledge Gaps on Biosimilars: Insights From Surveys*, 28 (2022), https://www.ajmc.com/view/biosimilarssuppl-insightssurveys (last visited Jul. 18, 2023).

23 US Food and Drug Administration, *Biosimilars Info Sheet: Biosimilar Regulatory Approval Pathway*, https://www.fda.gov/media/154914/download#:∼:text=Therefore%2C%20all%20biosimilar%20and%20interchangeable,result%20from%20the%20manufacturing%20process (accessed Sept. 2023).

24 Center for Biologics Evaluation and Research, *Scientific Considerations in Demonstrating Biosimilarity to a Reference Product, US Food and Drug Administration,*  https://www.fda.gov/media/82647/download (April 2015).

25 US Food and Drug Administration, *Biosimilars Info Sheet: Biosimilar Regulatory Approval Pathway*, https://www.fda.gov/media/154914/download#:∼:text=Therefore%2C%20all%20biosimilar%20and%20interchangeable,result%20from%20the%20manufacturing%20process (accessed Sept. 2023), *supra* note 22*.*

26 Michele K. Dougherty, Issam Zineh, and Leah Christl, *Perspectives on the Current State of the Biosimilar Regulatory Pathway in the United States*, 103 Clin. Pharm. Ther., no. 1 (2018): 36–38, https://doi.org/10.1002/cpt.909.

27
*The Complexities of Biosimilars and the Regulatory Approval Process*, accessed Jul. 21, 2022, https://www.ajmc.com/view/complexities-biosimilars-regulatory-approval-process.

28 Center for Biologics Evaluation and Research, *Long Term Follow-up After Administration of Human Gene Therapy Products*, U.S. Food and Drug Administration (2020), https://www.fda.gov/regulatory-information/search-fda-guidance-documents/long-term-follow-after-administration-human-gene-therapy-products (last visited Jun. 6, 2022).

29 Jote T. Bulcha et al., *Viral Vector Platforms within the Gene Therapy Landscape*, 6 Sig Transduct Target Ther 1 (2021).

30 Allogeneic vs. Autologous Treatments: Definitions and Differences, BioInformant (2022), http://https%253A%252F%252Fbioinformant.com%252Fallogeneic-versus-autologous%252F (last visited Jul. 21, 2022).

31 Center for Biologics Evaluation and Research, *Considerations for the Development of Chimeric Antigen Receptor (CAR) T Cell Products*, U.S. Food and Drug Administration (2022), https://www.fda.gov/regulatory-information/search-fda-guidance-documents/considerations-development-chimeric-antigen-receptor-car-t-cell-products (last visited Jul. 26, 2022).

32 Center for Drug Evaluation and Research, *Development of Therapeutic Protein Biosimilars: Comparative Analytical Assessment and Other Quality-Related Considerations Guidance for Industry*, U.S. Food and Drug Administration (2022), https://www.fda.gov/regulatory-information/search-fda-guidance-documents/development-therapeutic-protein-biosimilars-comparative-analytical-assessment-and-other-quality (last visited Sept. 5, 2023).

33 Office of the Commissioner, *Advancing Alternative Methods at FDA*, FDA (2022), https://www.fda.gov/science-research/about-science-research-fda/advancing-alternative-methods-fda (last visited Sept. 27, 2023).

34 Steven Oh, *Facilitating Advanced Technologies in Cell and Gene Therapies,* Presentation at CASSS, Jun. 7, 2021, https://www.casss.org/docs/default-source/cgtp/2021-cgtp-speaker-presentations/speaker-presentation-oh-steven-cber-fda-2021.pdf?sfvrsn=193414c_6.

35 Rachel Sher, *A Focal Point for FDA’s Rare Diseases Efforts: CDER’s New ARC Program*, https://advance.lexis.com/document/?pdmfid=1516831&crid=fab0a4fb-40c1-4757-b250-b4d9905d340f&pddocfullpath=%2Fshared%2Fdocument%2Fnews%2Furn%3AcontentItem%3A65GJ-9N41-F03R-N1M9-00000-00&pdcontentcomponentid=299488&pdteaserkey=sr1&pditab=allpods&ecomp=rz2yk&earg=sr1&prid=098bf71f-975b-4d70-939e-e44e2cb8b98b h (last visited Jul 21, 2022).

36 CITI Program, *FDA Announces New Accelerating Rare Disease Cures (ARC) Program | CITI Program*, https://about.citiprogram.org/, https://about.citiprogram.org/blog/fda-announces-new-accelerating-rare-disease-cures-arc-program/ (last visited Jul. 21, 2022).

37 Rachel Sher, *A Focal Point for FDA’s Rare Diseases Efforts: CDER’s New ARC Program*, *supra* note 32.

38 FDA, *Biosimilars Action Plan: Balancing Innovation and Competition* (2018) https://www.fda.gov/media/114574/download.

39 Center for Drug Evaluation and Research, Biosimilars | Science and Research, FDA (2023), https://www.fda.gov/drugs/biosimilars/biosimilars-science-and-research (last visited Apr 29, 2023).

40 Gerald Connolly, *HR 2617, Consolidated Appropriations Act, 2023* (2022), https://www.congress.gov/bill/117th-congress/house-bill/2617.

41 Joanne Eglovitch, *New OTP Head Discusses Policy Priorities for Cell and Gene Therapies, Regulatory Focus, A RAPS Publication*, https://www.raps.org/news-and-articles/news-articles/2023/9/new-otp-head-discusses-policy-priorities-for-cell.

42 Erin Harris, *Breaking Down Pricing Of Cell & Gene Therapies*, https://www.cellandgene.com/doc/breaking-down-pricing-of-cell-gene-therapies-0001 (last visited Mar. 20, 2023).

43 BioPharm, *Modular Manufacturing Platforms for Biologics,*  https://www.biopharminternational.com/view/modular-manufacturing-platforms-biologics (last visited Oct. 4, 2023).

44 Andelyn Biosciences, *Understanding The Gene Therapy Manufacturing Process*, https://andelynbio.com/2020/04/15/understanding-the-gene-therapy-manufacturing-process/ (Ap. 15, 2020).

45 Dan Wang, Phillip W. L. Tai & Guangping Gao, *Adeno-Associated Virus Vector as a Platform for Gene Therapy Delivery*, 18 Nat. Rev. Drug Discov. 358 (2019).

46
*Id*.

47 Novartis, *The Process of CAR-T Cell Therapy*, https://www.novartis.com/research-development/technology-platforms/cell-therapy/car-t-cell-therapy-and-beyond/car-t-healthcare-professionals/process-car-t-cell-therapy (accessed Jul. 26, 2022).

48 Genetic Engineering & Biotechnology News, *COG and Economies of Scale for Advanced Therapies*, https://www.genengnews.com/topics/bioprocessing/cog-and-economies-of-scale-for-advanced-therapies/ (2021).

49 Ruxandra-Maria Comisel et al., *Lentiviral Vector Bioprocess Economics for Cell and Gene Therapy Commercialization*, 167 Biochem. Eng. J. 107868 (2021).

50 For example, one 2018 analysis of the cost for manufacturing an autologous T-cell product found that 71% of COGS are from labor (the largest portion of which is from the manufacturing process): Katy Spink & Andrew Steinsapir, *The Long Road to Affordability: A Cost of Goods Analysis for an Autologous CAR-T Process*, 4 BioInsights 1105 (2018).

51 Richard P. Harrison et al., *Chimeric Antigen Receptor–T Cell Therapy Manufacturing: Modelling the Effect of Offshore Production on Aggregate Cost of Goods*, 21 Cytotherapy 224 (2019).

52 J. Fink, O. Karnieli, and D. Clarke, *ISCT Survey Reponses to Process and Product Development Committee’s Cost-of-Goods Questionnaire*, 21 Cytotherapy 5 Supplement (2019).

53 McKinsey, *Cell and Gene Therapy: Biopharma Portfolio Strategy,*  https://www.mckinsey.com/industries/life-sciences/our-insights/biopharma-portfolio-strategy-in-the-era-of-cell-and-gene-therapy (accessed Jul. 26, 2022).

54 Fierce Pharma, *$50 M Cell and Viral Vector Manufacturing Operation Backed by Harvard*, https://www.fiercepharma.com/manufacturing/50m-cell-and-viral-vector-manufacturing-operation-backed-by-harvard (2019).

55 US Pharmacopeia, *US Pharmacopeia Standards for Cell and Gene Therapy*, https://www.usp.org/sites/default/files/usp/document/our-work/biologics/asgct-poster.pdf (accessed Oct. 23, 2023).

56 US Food and Drug Administration, Standards Development for Regenerative Medicine Therapies, https://www.fda.gov/vaccines-blood-biologics/standards-development-regenerative-medicine-therapies (accessed Oct. 23, 2023)

57 Daniela Drago et al., *Global Regulatory Progress in Delivering on the Promise of Gene Therapies for Unmet Medical Needs*, 21 Mol. Ther. Methods Clin. Dev. 524 (2021).

58 Richard P. Harrison et al., *Decentralised Manufacturing of Cell and Gene Therapy Products: Learning from Other Healthcare Sectors*, 36 Biotechnol. Adv. 345 (2018).

59 R. Lee Buckler et al., *Technological Developments for Small-Scale Downstream Processing of Cell Therapies*, 18 Cytotherapy 301 (2016).

60
*Id.*

61 Genetic Engineering & Biotechnology News, *COG and Economies of Scale for Advanced Therapies*, https://www.genengnews.com/topics/bioprocessing/cog-and-economies-of-scale-for-advanced-therapies/ (2021).

62 Johannes C.M. van der Loo & J. Fraser Wright, *Progress and Challenges in Viral Vector Manufacturing*, 25 Hum. Mol. Genet. R42 (2016).

63 Karoline Hahn, *Decentralized Manufacturing: A Path towards Smart Cell and Gene Therapy Manufacturing*, MassBio, https://www.massbio.org/news/recent-news/decentralized-manufacturing-a-path-towards-smart-cell-and-gene-therapy-manufacturing/ (2022).

64 Christopher Reardon, Philip Vermilion, & John Yoshi Shyu, *Strategies for Scaling up and out in Gene Therapy Manufacturing: Addressing AAV’s Growing Pains*, BioInsights, https://insights.bio/cell-and-gene-therapy-insights/journal/article/2403/Strategies-for-scaling-up-and-out-in-gene-therapy-manufacturing-addressing-AAVs-growing-pains (2022).

65 A. D. Kaiser et al., *Towards a Commercial Process for the Manufacture of Genetically Modified T Cells for Therapy*, 22 Cancer Gene Ther. 72 (2015).

66 For example, pluripotent stem cells grow at a higher density than mesenchymal stem cells, and can produce 10x more cells through the manufacturing process: Richard P. Harrison, Nicholas Medcalf & Qasim A. Rafiq, *Cell Therapy-Processing Economics: Small-Scale Microfactories as a Stepping Stone toward Large-Scale Macrofactories*, 13 Regen. Med. 159 (2018).

67 The G-Rex cell culture platform provides the ability for large-scale cell expansion. Platform bioreactors are gas-permeable and membrane-based, so they do not need to actively delivery oxygen, and can hold a larger volume of nutrients to allow cells to reach maximum density: Josh Ludwig & Mark Hirschel, *Methods and Process Optimization for Large-Scale CAR T Expansion Using the G-Rex Cell Culture Platform*, 2086 Methods Mol. Biol. 165 (2020). Miniature bioreactors for cell-line development can also play a role in scale-up, as they provide high throughput for rapid screening. These bioreactors are also fully automated, and can be operated simultaneously to optimize production: Ruth C. Rowland-Jones et al., *Spectroscopy Integration to Miniature Bioreactors and Large Scale Production Bioreactors-Increasing Current Capabilities and Model Transfer*, 37 Biotechnol. Prog. e3074 (2021). In addition, stirred tank reactors have the potential to reduce COGS and facilitate scale-up as they are more widely adopted in cell therapy production: Richard P. Harrison, Nicholas Medcalf & Qasim A. Rafiq, *Cell Therapy-Processing Economics: Small-Scale Microfactories as a Stepping Stone toward Large-Scale Macrofactories*, 13 Regen. Med. 159 (2018).

68 Katy Spink & Andrew Steinsapir, *The Long Road to Affordability: A Cost of Goods Analysis for an Autologous CAR-T Process*, 4 BioInsights 1105 (2018).

69
*Id.*

70 P. Moutsatsou et al., *Automation in Cell and Gene Therapy Manufacturing: From Past to Future*, 41 Biotechnol. Lett. 1245 (2019).

71
*Id.*

72
*Id.*

73 Oliver Ball et al., *Bioprocessing Automation in Cell Therapy Manufacturing: Outcomes of Special Interest Group Automation Workshop*, 20 Cytotherapy 592 (2018). Existing cell therapy platforms have limited ability to provide real-time culture data besides pH and dissolved oxygen. These measures are important but fail to provide information on important characteristics like phenotype and functionality that developers can use to for platform adaptations. See: Matthew M. Hewitt et al., *Cell Therapy Manufacturing: Process Analytic Technologies Needed to Achieve Flexible, Feedback-Driven Automation*, 20 Curr. Opin. Biomed. Eng. 100, 358 (2021). Enhanced analytics can also facilitate the identification of critical process parameters (CPPs) and quality attributes of the manufacturing process to create a consistent, reproducible process and resulting product: Sean Chang, Bruce Greenwald, & Krishnendu Roy, *The Digital Revolution: Technological Innovations to Enable Automation in Cell Therapy Manufacturing*, 8 Cell Gene Ther. Insights 355 (2022). CPPs can additionally lower COGS by helping developers identify unnecessary production steps, identify batch failures earlier, and streamline the use of raw materials as needed, and in the manufacturing process, data analytics will also be important for the identification of multivariate parameters of a product that will impact patient outcomes.

74 Harrison et al, *Decentralised Manufacturing of Cell and Gene Therapy Products,* 36 Biotechnol. Adv. 345–357 (2018) https://www.sciencedirect.com/science/article/pii/S0734975017301647?via%3Dihub.

75 Karoline Hahn, *Decentralized Manufacturing: A Path towards Smart Cell and Gene Therapy Manufacturing*, MassBio, https://www.massbio.org/news/recent-news/decentralized-manufacturing-a-path-towards-smart-cell-and-gene-therapy-manufacturing/ (2022).

76
*Id.*

77 Emanuela M. Iancu & Lana E. Kandalaft, *Challenges and Advantages of Cell Therapy Manufacturing under Good Manufacturing Practices within the Hospital Setting*, 65 Curr. Opin. Biotechnol. 233 (2020).

78 Xiuyan Wang & Isabelle Rivière, *Clinical Manufacturing of CAR T Cells: Foundation of a Promising Therapy*, 3 Mol. Ther. Oncol. 16015 (2016).

79 Friends of Cancer Research, *Accelerating The Development of Engineered Cellular Therapies: A Framework for Extrapolating Data Across Related Products*, https://friendsofcancerresearch.org/wp-content/uploads/Accelerating_The_Development_of_Engineered_Cellular_Therapies.pdf?eType=EmailBlastContent&eId=f76efe17-9ea8-46e2-aa4b-977d2d19127b.

80 Arti K. Rai & W. Nicholson Price, *An Administrative Fix for Manufacturing Process Patent Thickets*, 39 Nat. Biotechnol. 20 (2021).

81 Rachel Goode and Bernard Chao, *Biological Patent Thickets and Delayed Access to Biosimilars, an American Problem,* 9 J. Law Biosci. (2022).

82 Victor L. van de Wiele, Aaron S. Kesselheim & Ameet Sarpatwari, *Barriers to US Biosimilar Market Growth: Lessons from Biosimilar Patent Litigation*, Health Aff. 1198, 1201 (2021).

83 Rachel Goode, William B. Feldman and Sean S. Tu, *Ancillary Product Patents to Extend Biologic Patent Life*, JAMA 330(21) (2023).

84 Jeffrey Wu and Claire Cheng, *Into the Woods: A Biologic Patent Thicket Analysis,* 19 Chi-Kent J. Intell. Prop. 94 (2020).

85 Arti K. Rai and W. Nicholson Price II, *An Administrative Fix for Manufacturing Process Patent Thickets,* 39 Nat. Biotechnol. 20–22 (2021).

86 Arti K. Rai, Rachel Sachs, and W. Nicholson Price II, *Cryptic Patent Reform Through the Inflation Reduction Act*, JOLT (2023).

87 Robin Feldman, *Trade Secrets in Biologic Medicine: The Boundary with Patents,* 24 STLR (2022).

88
*Id.*

89 W. Nicholson Price II and Arti Rai, *Manufacturing Barriers to Biologics Competition and Innovation*, 101 Iowa L. Rev. (2016).

90 Robin Feldman, *Trade Secrets in Biologic Medicine: The Boundary with Patents,* STLR (Forthcoming).

91 Price II and Arti Rai, *Manufacturing Barriers to Biologics Competition and Innovation*, 101 Iowa L. Rev. (2016), *supra* note 84.

92 Wuyuan Zhou and Xiang Wang, *Human Gene Therapy: A Patent Analysis*, 803 Gene (2021).

93 James F, Haley Jr, Karen Mangasarian, and Brian M Gummow, *Patent Issues in CAR-T Technology*, IAM, May 6, 2020 https://www.lexology.com/library/detail.aspx?g=b1b19489-bec8-4b50-9c37-cd9bb64081d7.

94 USPTO, *USPTO Fee Schedule*, https://www.uspto.gov/learning-and-resources/fees-and-payment/uspto-fee-schedule (last visited Oct. 4, 2023)

95 JB Fitzgerald and Jeffrey Morton, *Juno v. Kite Case Implications For Functionally Claimed Biological Compositions*, https://www.outsourcedpharma.com/doc/juno-v-kite-case-implications-for-functionally-claimed-biological-compositions-0001 (accessed Mar. 26, 2023).

96 35 U.S.C. § 112

97 USPTO, *Manual of Patent Examining Procedure (MPEP),*  https://www.uspto.gov/web/offices/pac/mpep/ (accessed Mar. 26, 2023).

98
*Juno Therapeutics, Inc. v. Kite Pharma, Inc.*, 2:17-cv-07639 (C.D. Cal.)

99
*Juno Therapeutics, Inc v. Kite Pharma, Inc.*, No. 20-1758 (Fed. Cir. 2021)

100 Amgen Inc. v. Sanofi Aventisub LL et al., Petition for a Writ of Certiorari, https://www.supremecourt.gov/DocketPDF/21/21-757/200548/20211118155938529_PCSK9%20Cert%20Petition%20-%20For%20Filing.pdf.

101 Ed Silverman, *A U.S. Court Ruling May Force Biologics Makers to Review Patent Protections,* STAT, Feb. 25, 2021, https://www.statnews.com/pharmalot/2021/02/25/amgen-sanofi-biologics-patents-lawsuit/.

102 See related: FDA, *Human Gene Therapy for Rare Diseases, Guidance for Industry*, January 2020, https://www.fda.gov/media/113807/download.

103 Benjamin N. Rome, ChangWon C. Lee, and Aaron S. Kesselheim, *Market Exclusivity Length for Drugs with New Generic or Biosimilar Competition, 2012–2018*, 109 Clin. Pharmacol. Ther. 367–71 (2021).

104 21 CFR **§** 316 (2023). https://public-inspection.federalregister.gov/2023-01179.pdf?utm_medium=email&utm_campaign=pi+subscription+mailing+list&utm_source=federalregister.gov.

105 21 CFR **§** 316 (2013).

106 21 CFR **§** 316 (2013)

107 FDA, *Interpreting Sameness of Gene Therapy Products Under the Orphan Drug Regulations, Guidance for Industry*, September 2021, https://www.fda.gov/media/134731/download.

108
*Catalyst Pharmaceuticals Inc. v. Becerra*, No. 20-13922 (D.D.C. Sept. 30, 2021)

109 See, e.g.: FDA, *Biosimilars and Interchangeable Biosimilars: Licensure for Fewer Than All Conditions of Use for Which the Reference Product Has Been Licensed Guidance for Industry*, https://www.fda.gov/regulatory-information/search-fda-guidance-documents/biosimilars-and-interchangeable-biosimilars-licensure-fewer-all-conditions-use-which-reference (accessed Apr. 9, 2020).

110 Although the CGT field is still relatively nascent, and thus extensive legislative prescriptions are beyond the scope of this early analysis, one could imagine a regime in which Congress granted somewhat longer regulatory exclusivities in exchange for originator disclosure of trade secrets.

111
*W.L. Gore & Assocs. v. Garlock, Inc.*, 721 F.2d 1540, (Fed. Cir.1983)

112 Dave CV Hartzema and Aaron Kesselheim, *Prices of Generic Drugs Associated With Numbers of Manufacturers*, New England J. Med. (2017).

113 BioMarin Pharmaceutical Inc, *US Food and Drug Administration Approves BioMarin’s ROCTAVIAN™ (Valoctocogene Roxaparvovec-Rvox), the First and Only Gene Therapy for Adults with Severe Hemophilia A,*  Cision, June 29, 2023, https://www.prnewswire.com/news-releases/us-food-and-drug-administration-approves-biomarins-roctavian-valoctocogene-roxaparvovec-rvox-the-first-and-only-gene-therapy-for-adults-with-severe-hemophilia-a-301867403.html.

114 See: Brian Canter et al., *Revisiting Interchangeability to Realize the Benefit of Biosimilars*, Duke Margolis, October 15, 2021, https://healthpolicy.duke.edu/publications/realizing-benefits-biosimilars-overcoming-rebate-walls.

115 FDA, *Biosimilarity and Interchangeability: Additional Draft Q&As on Biosimilar Development and BPCI Act- Guidance for Industry*, Biosimilars, September 2023, https://www.fda.gov/media/172169/download.

116 Nitzan Arad et al., *Realizing the Benefits of Biosimilars: Overcoming Rebate Walls*, Duke Margolis (2022) ^,^https://healthpolicy.duke.edu/publications/realizing-benefits-biosimilars-overcoming-rebate-walls.

117 The Social Security Act, 42 USC 1395ww **§** 1886(a)(4).

118 MedPAC, *Medicare Payment Strategies to Improve Price Competition and Value for Part B Drugs*, in Report to the Congress: Medicare and the Health Care Delivery System (2019) https://www.medpac.gov/wp-content/uploads/import_data/scrape_files/docs/default-source/reports/jun19_ch3_medpac_reporttocongress_sec.pdf.

119 See, Nitzan Arad et al., *Originator Biologics and Biosimilars: Payment Policy Solutions to Increase Price Competition While Maintaining Market Sustainability in Medicare Part B*, Duke Margolis (2021) https://healthpolicy.duke.edu/sites/default/files/2021-11/Realizing%20the%20Benefits%20of%20Biosimilars%20Part%20B.pdf.

120 Nitzan Arad et al., *Originator Biologics and Biosimilars: Payment Policy Solutions to Increase Price Competition While Maintaining Market Sustainability in Medicare Part B*, Duke Margolis (2021,) https://healthpolicy.duke.edu/sites/default/files/2021-11/Realizing%20the%20Benefits%20of%20Biosimilars%20Part%20B.pdf.; MedPAC*, Part B Drugs Payment Systems*, Paymentbasics (2022) https://www.medpac.gov/wp-content/uploads/2021/11/MedPAC_Payment_Basics_22_PartB_FINAL_SEC.pdf.

121 H.R.5376, 117th Congress (2021–2022)

122 Tarazi et al*, Medicare Beneficiary Enrollment Trends and Demographic Characteristics*, HHS ASPE Office Oo Health Policy Issue Brief (2022) https://aspe.hhs.gov/sites/default/files/documents/f81aafbba0b331c71c6e8bc66512e25d/medicare-beneficiary-enrollment-ib.pdf.

123 CMS Administrator Chiquita Brookes-LaSure*, Medicare Drug Price Negotiation Program: Next Steps in Implementation for Initial Price Applicability Year 2026*, January 11, 2023, https://www.cms.gov/files/document/medicare-drug-price-negotiation-program-next-steps-implementation-2026.pdf.

124 G. Doug Myers et al*.*, *Perspectives on Outpatient Administration of CAR-T Cell Therapy in Aggressive B-Cell Lymphoma and Acute Lymphoblastic Leukemia*, 9 J. Immunother Cancer (2021).

125 Centers for Medicare and Medicaid Services, *Medicare Drug Price Negotiation Program: Revised Guidance, Implementation of Sections 1191–1198 of the Social Security Act for Initial Price Applicability Year 2026*, June 30, 2023, https://www.cms.gov/files/document/revised-medicare-drug-price-negotiation-program-guidance-june-2023.pdf.

126 Memorandum from Meena Seshamani to Interested Parties (June 30, 2023) (on file with the Center for Medicare and Medicaid Services)

127 Nitzan Arad and Mark B. McClellan, *Drug Pricing Reform in the Inflation Reduction Act: What Are the Implications? Part 2*, Health Aff., December 15, 2022.

128 Arti K. Rai, Rachel Sachs, and W. Nicholson Price II, *Cryptic Patent Reform Through the Inflation Reduction Act*, JOLT (2023).

129 Inmaculada Hernandez and Walid F. Gellad, *Differences between Managed Care and Fee for Service Medicaid in the Use of Generics for High-Rebate Drugs: The Cases of Insulin Glargine and Glatiramer*, 26 J. Manag. Care Spec. Pharm. 154–59 (2020).

130
*Id.*

131 Rachel Garfield, Rachel Dolan, and Elizabeth Williams*, Costs and Savings under Federal Policy Approaches to Address Medicaid Prescription Drug Spending*, KFF, June 22, 2021, https://www.kff.org/medicaid/issue-brief/costs-and-savings-under-federal-policy-approaches-to-address-medicaid-prescription-drug-spending/.

132
*Supra* note 129.

133 Arad, Nitzan et al., *Realizing The Benefits OF Biosimilars: What the U.S. Can Learn from Europe*, Duke Margolis (2021) https://healthpolicy.duke.edu/publications/realizing-benefits-biosimilars-what-us-can-learn-europe.

134 Va. Code § 54.1–3442.02 (2021) https://law.lis.virginia.gov/vacode/54.1-3442.02/.

135 President Joseph Biden, *Executive Order on Lowering Prescription Drug Costs for Americans,* The White House, October 14, 2022 https://www.whitehouse.gov/briefing-room/presidential-actions/2022/10/14/executive-order-on-lowering-prescription-drug-costs-for-americans/.

136 Assistant Secretary for Public Affairs (ASPA), *HHS Secretary Responds to the President’s Executive Order on Drug Prices*, US Department of Health And Human Services, February 14, 2023, https://www.hhs.gov/about/news/2023/02/14/hhs-secretary-responds-to-the-presidents-executive-order-on-drug-prices.html.

137
*Id*.

138 CAR-T has demonstrated that the unpredictability of the cost of treatment is another factor making it difficult for Medicaid reimbursement because actuaries are unable to know if patients will be low or high cost, worrying that the costs will exceed the bundled payment amount. This contributed to hurdles in the rollout of CAR-T in Medicaid because not all children’s hospitals developed the expertise in CAR-T to bring down the costs and sustainably provide CAR-T therapy.

139 Namely, Bluebird Bio, CRISPR and Vertex, and Intellia, although a few companies have also ceased development of their gene therapies for SCD in 2023

140 Allison DeAngelis, *Bluebird Submits Sickle Cell Gene Therapy to FDA for Approval*, STAT (blog), April 24, 2023, https://www.statnews.com/2023/04/24/bluebird-submits-sickle-cell-gene-therapy-to-fda-for-approval/; BioPharma, *Dive Sickle Cell Pipeline Narrows as Gene Therapy Developers Rethink Research Plans,*  https://www.biopharmadive.com/news/intellia-graphite-sangamo-sickle-cell-gene-therapy-discontinue/643400/ (accessed May 30, 2020); Max Bayer, *Sangamo Shelves Sickle Cell Therapy, Joining Rivals Forced to Rethink Development Plans*, Fierce Biotech., February 23, 2023.

141 Bhuiyan Khan, Beena et al., *Performance Measurement Strategies in Value-Based Payment Arrangements,*  Duke Margolis (2023) https://healthpolicy.duke.edu/publications/performance-measurement-strategies-value-based-payment-arrangements, though this is something CMS is hoping to address through its Innovation Center’s CGT Access Model

142 Cigna, *Embarc Benefit Protection*, https://www.cigna.com/employers-brokers/cost-control/embarc-benefit-protection (accessed Jul. 21, 2023).

143 Paige Minemyer, *Cigna Launches New Benefits Solution Aimed at Making Gene Therapy More Affordable*, Fierce Healthcare, September 6, 2019, https://www.fiercehealthcare.com/payer/cigna-launches-new-benefits-solution-aimed-at-making-gene-therapy-more-affordable.

